# Efficient production of cembratriene-ol in *Escherichia coli* via systematic optimization

**DOI:** 10.1186/s12934-023-02022-4

**Published:** 2023-01-24

**Authors:** Haiquan Yang, Kunjie Zhang, Wei Shen, Lei Chen, Yuanyuan Xia, Wei Zou, Yu Cao, Xianzhong Chen

**Affiliations:** 1 The Key Laboratory of Carbohydrate Chemistry and Biotechnology, School of Biotechnology, Ministry of Education, Jiangnan University, 214122 Wuxi, China; 2 The Key Laboratory of Industrial Biotechnology, School of Biotechnology, Ministry of Education, Jiangnan University, 214122 Wuxi, China; 3 College of Bioengineering, Sichuan University of Science & Engineering, 644000 Yibin, Sichuan China

**Keywords:** *Escherichia coli*, DXP pathway, Cembratriene-ol, Key enzymes, Systematicoptimization

## Abstract

**Background:**

The tobacco leaf–derived cembratriene-ol exhibits anti-insect effects, but its content in plants is scarce. Cembratriene-ol is difficult and inefficiently chemically synthesised due to its complex structure. Moreover, the titer of reported recombinant hosts producing cembratriene-ol was low and cannot be applied to industrial production.

**Results:**

In this study, *Pantoea ananatis* geranylgeranyl diphosphate synthase (CrtE) and *Nicotiana tabacum* cembratriene-ol synthase (CBTS) were heterologously expressed to synthsize the cembratriene-ol in *Escherichia coli*. Overexpression of cbts*, the 1-deoxy-d-xylulose 5-phosphate synthase gene *dxs*, and isopentenyl diphosphate isomerase gene *idi* promoted the production of cembratriene-ol. The cembratriene-ol titer was 1.53-folds higher than that of *E*. *coli* Z17 due to the systematic regulation of *ggpps*, cbts*, *dxs*, and *idi* expression. The production of cembratriene-ol was boosted via the overexpression of genes *ispA*, *ispD*, and *ispF*. The production level of cembratriene-ol in the optimal medium at 72 h was 8.55-folds higher than that before fermentation optimisation. The cembratriene-ol titer in the 15-L fermenter reached 371.2 mg L^− 1^, which was the highest titer reported.

**Conclusion:**

In this study, the production of cembratriene-ol in *E*. *coli* was significantly enhanced via systematic optimization. It was suggested that the recombinant *E*. *coli* producing cembratriene-ol constructed in this study has potential for industrial production and applications.

**Supplementary Information:**

The online version contains supplementary material available at 10.1186/s12934-023-02022-4.

## Background

Natural products include terpenoids, polyketides, phenylpropanoids, alkaloids, and so on [1]. Terpenoids and their derivatives are compounds obtained from mevalonic acid with the isoprene unit (C5) as the basic structural unit in the molecular skeleton [2]. Due to their unique and diverse bioactive characteristics, terpenoids play an important role in fields and applications such as pharmaceuticals, spices, food, biofuels, cosmetics, and industrial raw materials [3–6], which have attracted the interest of many researchers. Cembratriene-ol is a monocyclic diterpenoid that exhibits good insect deterrent effects [7–11]. This compound is a plant natural product that is mainly found in *Nicotiana* plants [7], and it can be completely degraded in the natural environment without residue, making it a sustainable biopesticide ingredient [12, 13]. The titer of natural products extracted from plants cannot commonly meet the demands of their industrial application due to the lack of efficient production methods [1, 14]. The content of cembratriene-ol in plants is low (0.18% of the leaf dry weight) [10, 15], and direct extraction of cembratriene-ol from plants is difficult and expensive [15]. Moreover, cembratriene-ol is difficult and inefficiently chemically synthesised due to its complex structure [16]. The total chemical synthesis of plant natural products commonly produces significant waste, which may lead to further pollution [17].

Heterologous production pathways of natural chemicals in their natural production hosts can be transplanted or employed to construct engineered microbial cells producing these natural chemicals [1]. The biosynthesis of plant natural products with high value by engineered microbial cell factories has great prospects for their industrial production and application and provides a prospective solution for protecting plants and land and averting pollution from chemical synthesis [14, 18, 19]. Many studies have reported on heterologous synthesis of plant-based products in engineered microbial cell factories, such as cannabinoids [20], isoprenoids [21], and glycyrrhetinic acid [22]. Genetic and metabolic engineering methods have been used to optimize and modify metabolic pathways of microbial cells to efficiently produce high value chemicals [23]. Sophisticated metabolic engineering approaches can be employed to redirect the metabolic flux of microbial cells toward the formation of target products [1]. Moreover, systems metabolic engineering methods can be performed to construct an efficient microbial cell factory by generating elucidating metabolic processes and integrating these techniques with genetic engineering, synthetic biology, and systems biology [24–27].

**Fig. 1:**
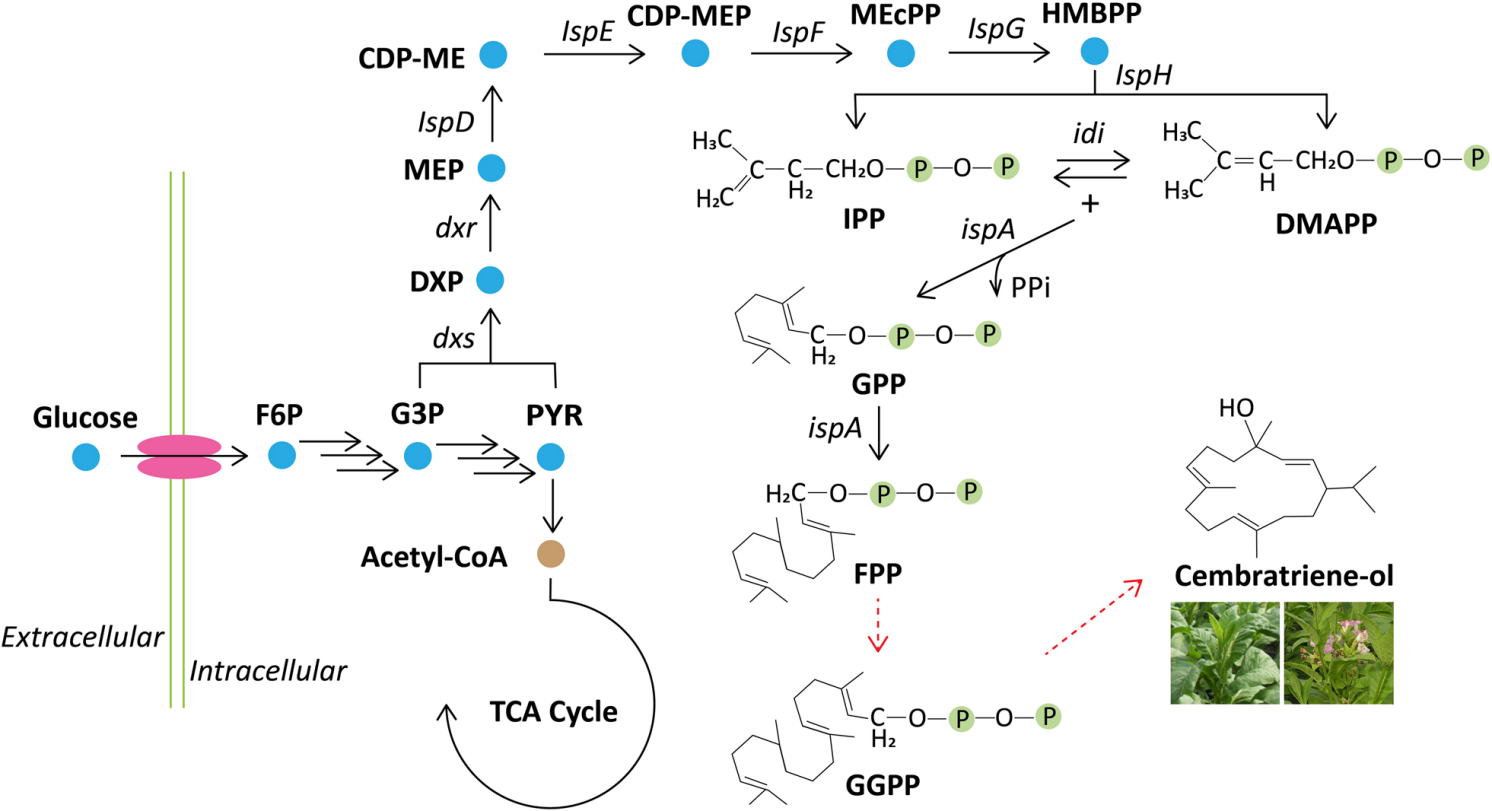
Synthetic pathway of cembratriene-ol in *E. coli*. F6P, fructose 6-phosphate; G3P, glyceraldehyde 3-phosphate; PYR, pyruvate; DXP, 1-deoxy-d-xylulose-5-phosphate; MEP, 2-*C*-methyl-d-erythritol-4-phosphate; CDP-ME, 4-(cytidine 5’-diphospho)-2-*C*-methyl-d-erythritol; CDP-MEP, 2-phospho-4-(cythidine 5’-diphospho)-2-*C*-methyl-d-erythritol; MEcPP, 2-C-methyl-d-erythritol 2,4-cyclodiphosphate; HMBPP, 1-hydroxy-2-methyl-2-butenyl 4-diphosphate; IPP, 4-hydroxyphenylpyruvate; DMAPP, dimethylallyl diphosphate; GPP, geranyl diphosphate; FPP, farnesyl diphosphate; GGPP, geranylgeranyl diphosphate; *dxs*, 1-deoxy-d-xylulose 5-phosphate synthase gene; *dxr*, 1-deoxy-d-xylulose 5-phosphate reductoisomerase gene; *IspD*, 2-*C*-methyl-d-erythritol 4-phosphate cytidylyltransferase gene; *IspE*, 4-(cytidine 5’-diphospho)-2-*C*-methyl-d-erythritol kinase gene; *IspF*, 2-*C*-methyl-d-erythritol 2,4-cyclodiphosphate synthase gene; *IspG*, (*E*)-4-hydroxy-3-methylbut-2-enyl-diphosphate synthase gene; *IspH*, 1-hydroxy-2-methyl-2-(*E*)-butenyl 4-diphosphate reductase; *idi*, isopentenyl-diphosphate Δ-isomerase gene; *ispA*, geranyl diphosphate synthase gene

With the continuous advances in biotechnology, an increasing number of microorganisms have been developed and modified to produce high-value chemicals, such as *Escherichia coli* [28, 29], *Saccharomyces cerevisiae* [30, 31], and *Bacillus subtilis* [32]. Among them, *E*. *coli* is one of the most commonly used foundational strains for the production of natural compounds, which includes many advantages (e.g. high growth rate and cell density culture, and well-established metabolic engineering tools and methods) [1, 28]. *E. coli* includes the natural 1-deoxy-d-xylulose-5-phosphate (DXP) pathway [1], which provides the precursor (farnesyl pyrophosphate, FPP) for synthesising cembratriene-ol from plants (Fig. 1). The overexpression of rate-limiting enzymes in the DXP pathway promotes carbon metabolic flux to the target products [1].

In this study, we overexpressed the farnesyl diphosphate synthase gene *ispA*, the 2-C-methyl-d-erythritol 4-phosphate cytidylyltransferase gene *ispD*, 2-C-methyl-d-erythritol 2,4-cyclodiphosphate synthase gene *ispF*, and 1-deoxy-d-xylulose-5-phosphate synthase gene *dxs* from *E*. *coli*, isopentenyl diphosphate isomerase gene *idi* from *Haematococcus lacustris*, geranylgeranyl pyrophosphate synthetase (CrtE) gene *ggpps* from *Pantoea ananatis*, and cembratriene-ol synthase gene cbts* from *Nicotiana tabacum* in recombinant *E*. *coli* and systematically regulated their expession levels to boost the titer of cembratriene-ol. Introduction of the heterologous mevalonate (MVA) pathway from *Streptomyces* and fermentation optimisation methods and a 15-L fermenter were also used to enhance the titer of cembratriene-ol. Moreover, the productivity, conversion yield, cell growth, residual glucose, and other parameters were determined and analysed.

## **Results and discussion**

### **Construction of recombinant*E. coli*producing cembratriene-ol**

**Fig. 2:**
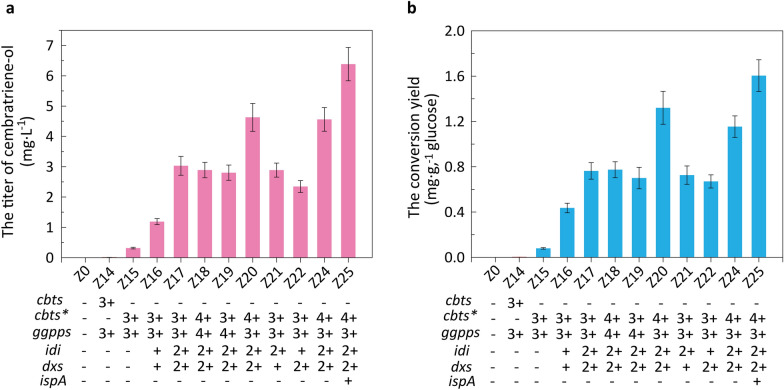
Construction of recombinant strains producing cembratriene-ol and its efficient production via optimizing key enzyme expression level. **a** The titer of cembratriene-ol; **b** Residual glucose concentration and conversion yield of glucose to cembratriene-ol. cbts* lacked a plastid transit peptide (52 AA, 156 bp) compared with *cbts*; *ggpps*, geranylgeranyl pyrophosphate synthase gene; *idi*, isopentenyl-diphosphate Δ-isomerase gene; *dxs*, 1-deoxy-d-xylulose 5-phosphate synthase gene; *ispA*, geranyl diphosphate synthase gene. −, no gene overexpression; +, gene overexpression using pACYCDuet-1; 2+, gene overexpression using pCDFDuet-1; 3+, gene overexpression using pETDuet-1; 4+, gene overexpression using pRSFDuet-1.

*E. coli* contains the natural DXP pathway, which can synthesise terpenoid skeleton chemicals. This pathway includes seven enzyme-catalysed reaction steps and requires NADPH and ATP [1]. Farnesyl pyrophosphate (FPP) can be synthesised and accumulated via the natural DXP pathway in *E. coli*. Based on the initial product FPP, cembratriene-ol can be produced via heterologous expression of geranylgeranyl pyrophosphate synthase and cembratriene-ol synthase in *E*. *coli* [12]. In this study, the geranylgeranyl pyrophosphate synthase gene *ggpps* and cembratriene-ol synthase gene *cbts* were heterologously expressed to synthesize cembratriene-ol in *E. coli* (Additional file 1: Fig. S1/S2/S3a). As shown in Fig. 2a, recombinant *E. coli* Z14 was constructed, with a titer of 1.58 × 10^− 2^ mg L^− 1^. The control strain without *ggpps* and *cbts* genes could not produce cembratriene-ol. Genes *ggpps* and *cbts* were expressed in *E. coli*, and cembratriene-ol was successfully synthesised. The productivity and conversion yield of glucose to cembratriene-ol in Z14 were 3.3 × 10^− 4^ mg L^− 1^·h^− 1^ and 0.04 mg g^− 1^ at 48 h, respectively (Fig. 2b, Additional file 1: Fig. S4). Since the growth rate is one of significant factors for the specific productivity [33], the cell growth and growth rate were determined in this study. The maximum cell density (OD_600_) and specific growth rate of Z14 were 3.73 and 0.24 h^− 1^, which of the control was 3.65 and 0.27 h^− 1^, respectively (Additional file 1: Fig. S3b/c). In the previous study, the cembratriene-ol toxicity effects were not observed for *E*. *coli* at the experimental concentration (0.25%) [12].

### Effect of the cembratriene-ol synthase and its truncated variant on cembratriene-ol production

As a key enzyme in the synthesis of cembratriene-ol, cembratriene-ol synthase is of great significance for the production of cembratriene-ol in recombinant *E*. *coli*. Mischko et al. expressed one cembratriene-ol synthase lacking the plastid transit peptide to synthesize cembratriene-ol [12]. In this study, the effects of the cembratriene-ol synthase gene *cbts* and its truncated variant cbts* on the synthesis of cembratriene-ol in recombinant *E. coli* were compared, and the recombinant *E. coli* Z14 and *E*. *coli* Z15 were constructed (Additional file 1: Fig. S1/S5a). The cembratriene-ol titer and productivity in *E*. *coli* Z15 expressing cbts* were increased by 20-folds higher than those of *E. coli* Z14 (Fig. 2a). The cbts* gene was also found to be more conducive to synthesizing cembratriene-ol in recombinant *E. coli* than *cbts*. It was found that the solubility of enzyme CBTS with plastid transit peptide sequence was reduced compared with CBTS* when they were heterologously overexpressed in *E*. *coli* (Additional file 1: Fig. S6). The conversion yield of glucose to cembratriene-ol in *E*. *coli* Z15 was 2-folds higher than that of *E. coli* Z14, indicating that the overexpression of cbts* facilitates the conversion of glucose to cembratriene-ol. The growth of *E*. *coli* Z15 was inhibited by the expression of cbts*, and the main reason may be that the large amount of glucose was used for synthesis of cembratriene-ol instead of cell growth (Additional file 1: Fig. S5b/c).

### Effect of overexpressing key genes *dxs* and *idi* in carbon metabolism flux on cembratriene-ol production

The supply of isoprene pyrophosphate (IPP) and dimethylallyl diphosphate (DMAPP) is very important for the production of cembratriene-ol [12]. The 1-deoxy-d-xylulose 5-phosphate synthase DXS (product of the *dxs* gene) and IPP isomerase IDI (product of the *idi* gene) are two key rate-controlling enzymes in the DXP pathway of *E. coli*. The gene *idi* plays a significant role in controlling the isoprenoid flux [33]. Park et al. overexpressed IDI and geranylgeranyl diphosphate synthase GPS from *Archaeoglobus fulgidus* to produce astaxanthin in an engineered *E*. *coli* [34]. Genes *dxs* and *idi* were co-expressed using plasmid pACYCDuet-1 in *E*. *coli* Z15 to construct recombinant *E*. *coli* Z16 (Additional file 1: Fig. S1/S7a). The titer (1.19 mg L^− 1^) of cembratriene-ol in *E*. *coli* Z16 was increased by 3.75-folds that of *E*. *coli* Z15 (Fig. 2a). The productivity of cembratriene-ol in *E*. *coli* Z16 was increased from 6.6 × 10^− 3^ mg L^− 1^·h^− 1^ in *E*. *coli* Z15 to 2.5 × 10^− 2^ mg L^− 1^·h^− 1^, which indicated that overexpression of *dxs* and *idi* increased the carbon metabolism flux of the DXP pathway to cembratriene-ol and further enhanced the production of cembratriene-ol in recombinant *E. coli*. It was also reported that genes *dxs* and *idi* were overexpressed to improve the production of heterologous terpene (e.g. cembratriene-ol) [12, 35]. The growth ability of *E*. *coli* Z16 was inhibited to a certain extent compared with *E*. *coli* Z15, suggesting that overexpression of *dxs* and *idi* increased the flux of carbon metabolism to cembratriene-ol but not cell growth (Additional file 1: Fig. S7b/c). *E*. *coli* Z16 had a higher residual glucose concentration (1.27 g L^− 1^) in fermentation liquid compared with that of *E*. *coli* Z15, and the conversion yield of glucose to cembratriene-ol was 5.5-folds that of *E*. *coli* Z15 (Fig. 2b). Overexpression of *dxs* and *idi* was further verified to improve the conversion efficiency of glucose to cembratriene-ol via the DXP pathway in *E. coli*.

To further enhance the production of cembratriene-ol, plasmid pCDFDuet-1 was used to increase the expression levels of *idi* and *dxs*, and the recombinant *E*. *coli* Z17 was constructed (Additional file 1: Fig. S1/S8a). The titer and productivity of cembratriene-ol of *E*. *coli* Z17 reached 3.03 mg L^− 1^ and 6.3 × 10^− 2^ mg L^− 1^·h^− 1^, respectively, which was increased by 2.6-folds that of *E*. *coli* Z16 (Fig. 2). It was suggested that enhancing the expression levels of *idi* and *dxs* was beneficial for the efficient production of cembratriene-ol by recombinant *E. coli*. Yuan et al. also found a medium-copy plasmid pDCQ108 was used to express key enzymes (e.g., *idi* and *dxs*) to result in a significant increase in production of β-carotene compared with that of the low-copy plasmid pPCB15 [33]. Cell growth of *E*. *coli* Z17 was inhibited to a certain extent compared with *E*. *coli* Z16 (Additional file 1: Fig. S8b/c), and the carbon metabolism flux in the DXP pathway to cembratriene-ol should be one of the main reasons. The residual glucose concentration in the fermentation liquid of *E*. *coli* Z17 was close to 0, and the conversion yield of glucose to cembratriene-ol reached 0.763 mg g^− 1^, which was 1.75-folds that of *E*. *coli* Z16. The enhanced expression levels of *idi* and *dxs* were beneficial for converting glucose to cembratriene-ol. Plasmid pRSFDuet-1 with a high copy number was used to increase the expression levels of *ggpps* and cbts* in *E*. *coli* Z17 for efficient synthesis of cembratriene-ol, and recombinant *E*. *coli* Z18 was constructed (Additional file 1: Fig. S8a). The titer and productivity of cembratriene-ol in *E*. *coli* Z18 was not significantly improved, and it was indicated that overexpression of *ggpps* and cbts* under these conditions could not yield high efficiency cembratriene-ol production.

### Enhancing cembratriene-ol production via systematic regulation of key gene expression

The DXP pathway contains multiple complex regulatory elements that are difficult to systematically optimise to enhance precursor accumulation (e.g. FPP). The accumulation of sufficient precursors plays an important role in producing the target compounds. Enhancing the biosynthetic flux of precursors in the DXP pathway in *E. coli* is a key strategy for increasing the accumulation of target products [1]. Based on the above results, it was found that the different expression levels of key genes *idi*, *dxs*, *ggpps*, and cbts* have an important influence on cembratriene-ol production in *E. coli*. The plasmids pACYCDuet-1, pCDFDuet-1, pETDuet-1, and pRSFDuet-1 were used to regulate the expression levels of key genes *idi*, *dxs*, *ggpps*, and cbts*, and four recombinant *E. coli* Z19, *E*. *coli* Z20, *E*. *coli* Z21, and *E*. *coli* Z22 were constructed (Additional file 1: Fig. S1/S9a). The theoretical copy numbers of plasmids pACYCDuet-1, pCDFDuet-1, pETDuet-1, and pRSFDuet-1 are 10, 20, 40, and 100, respectively [36]. In this study, the actual copy numbers of empty plasmids pACYCDuet-1, pCDFDuet-1, pETDuet-1, and pRSFDuet-1 are 3.38, 4.89, 9.12, and 26.3 at the cultivation conditions, respectively (Additional file 1: Fig. S10). The titers of cembratriene-ol in *E*. *coli* Z19, *E*. *coli* Z20, *E*. *coli* Z21, and *E*. *coli* Z22 were 2.80, 4.63, 2.89, and 2.35 mg L^− 1^, respectively (Fig. 2a). The titer of cembratriene-ol in *E*. *coli* Z20 was the highest, exhibiting an increase by 1.53-folds compared with that of *E*. *coli* Z17. The productivity of cembratriene-ol in *E*. *coli* Z20 was 9.6 × 10^− 2^ mg L^− 1^·h^− 1^, which was significantly higher than that of *E*. *coli* Z17. *E*. *coli* Z20 had the highest conversion yield of glucose to cembratriene-ol (1.32 mg g^− 1^). It was presumed that this key enzyme expression mode (pCDFDuet-1 with *idi* and *dxs*, pRSFDuet-1 with cbts*, and pETDuet-1 with *ggpps*) is beneficial for converting glucose to cembratriene-ol. The systemic regulation of *idi*, *dxs*, *ggpps*, and cbts* expression levels inhibited cell growth compared to that of the control (Additional file 1: Fig. S9b/c). The order of gene expression levels for improving the synthesis ability of cembratriene-ol in *E*. *coli* was cbts* > *ggpps* > *idi* > *dxs*, suggesting that overexpression of cbts* and *ggpps* using plasmids with high copies was preferred for the efficient synthesis of cembratriene-ol. These results indicated that cbts* and *ggpps* were critical genes for the efficient synthesis of cembratriene-ol in *E*. *coli*. Wang et al. also found that geranylgeranyl diphosphate synthase was a major rate-limiting enzyme for the biosynthesis of isoprenoids in engineered *E*. *coli* [34, 36].

### Enhancing cembratriene-ol production by overexpressing the farnesyl diphosphate synthase gene *ispA*

Farnesyl diphosphate synthase ispA synthesising GPP and FPP significantly contributes to the biosynthetic system of the terpenoid carbon skeleton [38]. In this work, the recombinant *E*. *coli* Z25 was constructed via transforming the recombinant plasmid pACYCDuet-1-*ispA* into *E*. *coli* Z20 (Additional file 1: Fig. S1/S11a). The titer of cembratriene-ol in *E*. *coli* Z25 was significantly improved compared to that of *E*. *coli* Z24 (Fig. 2a). The titer and productivity of cembratriene-ol in *E*. *coli* Z25 were also enhanced compared those of *E*. *coli* Z24. The overexpression of farnesyl diphosphate synthase ispA significantly enhanced the synthesis of cembratriene-ol in *E. coli*. Meanwhile, cembratriene-ol was also efficiently synthesised in *S*. *cerevisiae* BY-T20 with high production level of geranylgeranyl pyrophosphate (GGPP) via enhancing MVA pathway, the titer of which reached approximately 1.56 mg L^− 1^ [39]. The glucose consumption of *E*. *coli* Z25 was close to that of *E*. *coli* Z24, and cell growth was not inhibited compared with *E*. *coli* Z24 (Additional file 1: Fig. S11b/c). *ispA* overexpression has little effect on the glucose consumption and cell growth of recombinant *E. coli*.

### Enhancing the production of cembratriene-ol via optimised fermentation

**Fig. 3:**
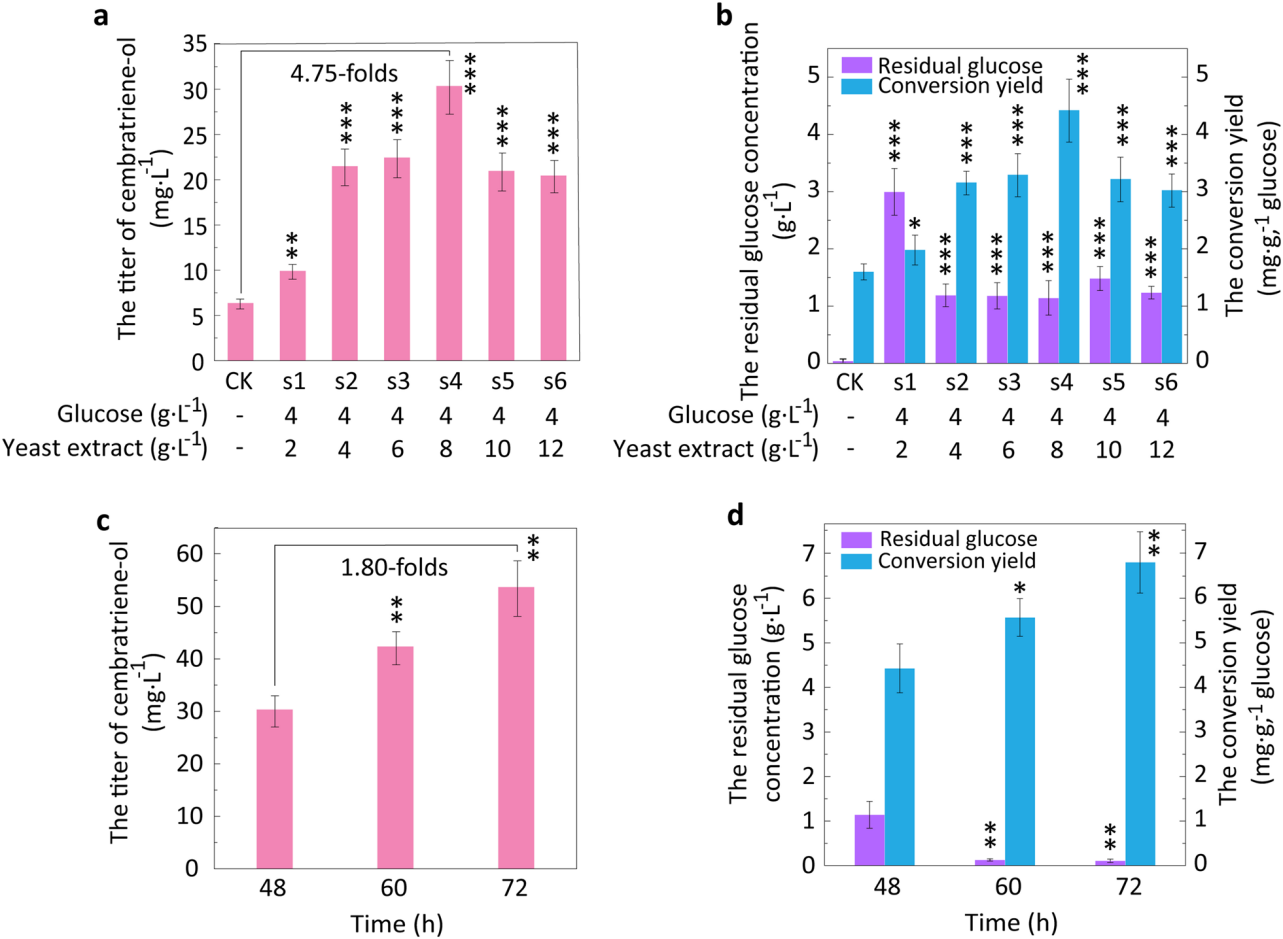
Effects of fermentation optimization on production of cembratriene-ol. **a** Effects of adding glucose and yeast extract with different proportions on the production of cembratriene-ol. **b** Effects of adding glucose and yeast extract with different proportions on the residual glucose concentration and the conversion yield at 48 h. CK means no addition of additional glucose and yeast extract, and the glucose concentration of CK is 4 g L^-1^. **c** Effects of different fermentation time on cembratriene-ol production under the proportion of glucose:yeast extract = 4:8. **d** Effects of different fermentation time on the residual glucose concentration and the conversion yield. Student’s *t*-test was used to statistically analyze data, and statistical significance was set at *p* value < 0.05. Asterisks indicate significant differences compared to *E*. *coli* Z25 (***, *p* < 0.01; **, *p* < 0.05; *, *p* > 0.05). *p* < 0.05 was considered statistically significant

Besides engineering strategies focus on biosynthetic pathways, the microbial cell factories performance can be greatly enhanced by appropriate optimization of the fermentation conditions [40]. Fermentation processes have been optimised to further improve the production of target products in recombinant *E*. *coli*, such as medium optimisation and environmental parameter optimisation. Carbon source and nitrogen source, as important medium components, play crucial roles in the production of primary and secondary metabolites and the cell growth [41]. The production level of cembratriene-ol was further improved by adding different proportions of glucose and yeast extract (Fig. 3). When different proportions of added glucose and yeast extract were 4:2, 4:4, 4:6, 4:8, 4:10, and 4:12, the titers of cembratriene-ol in *E*. *coli* Z25 were 9.94, 21.51, 22.47, 30.37, 21.00, and 20.48 mg L^− 1^, respectively. When the proportion of added glucose and yeast extract was 4:8, the titer, productivity, and conversion yield of glucose to cembratriene-ol were the highest, increasing by 4.75-, 4.75-, and 2.75-folds that of the control (without addition of glucose and yeast extract), respectively. It was indicated that high nitrogen source concentration promoted production of cembratriene-ol. It was also found that the addition of nitrogen source with high concentrations was advantageous for efficient accumulation of rhamnolipids [41, 42]. There was residual glucose when glucose and yeast extract were supplemented. When the proportion of added glucose and yeast extract was 4:2, the residual glucose concentration was the highest (up to 2.99 g L^− 1^). High concentrations of glucose cannot be fully utilised compared to yeast extract at high concentrations. The synthesis of primary or secondary metabolites and/or the cell growth can be usually influenced by the metabolic rate of carbon sources [41]. The growth of recombinant *E*. *coli* was significantly improved by the addition of glucose and yeast extract in different proportions compared with that of the control (without addition of glucose and yeast extract). When the proportion of added glucose and yeast extract was 4:8, the cell density was the highest and the cell growth was the fastest. The maximum OD_600_ and the maximum specific growth rate were increased by 3.00- and 3.23-folds those of the control (without addition of glucose and yeast extract), respectively (Additional file 1: Fig. S12a/b). However, when the proportion of added glucose and yeast extract was 4:2, the cell growth was lower than those of other proportions of added glucose and yeast extract. It was also found that astaxanthin production in *Xanthopyllomyces dendrorhous* could be promoted by high C/N, but the cell growth could be inhibited by high glucose [40]. Meanwhile, the pH changes of fermentation broth at different C/N ratios were determined, and the pH values at 4:8, 4:10, and 4:12 (C/N ratios) were 6.55, 6.60, and 6.64 at 48 h, respectively. Nitrogen sources can promote cell growth of microorganisms. Too many nitrogen sources can make the cell row too vigorously and the pH value is too high, which is not conducive to the accumulation of metabolites. Improper C/N ratios also affect the proportion of nutrients absorbed by the cell, directly affecting the cell growth and the product synthesis.

The above results showed that glucose could not be completely utilised by recombinant *E*. *coli*. The concentration of residual glucose reached 1.14 g L^− 1^ at 48 h when the proportion of added glucose and yeast extract was 4:8. It was presumed that the extension of the fermentation period could increase the production of cembratriene-ol in *E*. *coli* Z25. In this study, the fermentation period was optimised to improve cembratriene-ol production. As shown in Fig. 3c, the cembratriene-ol production by *E*. *coli* Z25 was further enhanced during the extension of fermentation time. The titers of cembratriene-ol reached 42.13 and 53.72 mg  L^− 1^ at 60 and 72 h, respectively (Fig. 3c). The production intensities of cembratriene-ol also increased to 0.70 and 0.75 mg  L^− 1^ h^− 1^ at 60 and 72 h, respectively (Fig. 3d). The titer of cembratriene-ol at 72 h was 1.8-folds higher than that at 48 h and 3357.5-folds that of the initially constructed *E*. *coli* Z14. The conversion yield of glucose to cembratriene-ol reached 6.81 mg g^− 1^ at 72 h. It was presumed that glucose was gradually converted into cembratriene-ol during extension of fermentation time.

### Introduction of the heterologous MVA pathway

**Fig. 4:**
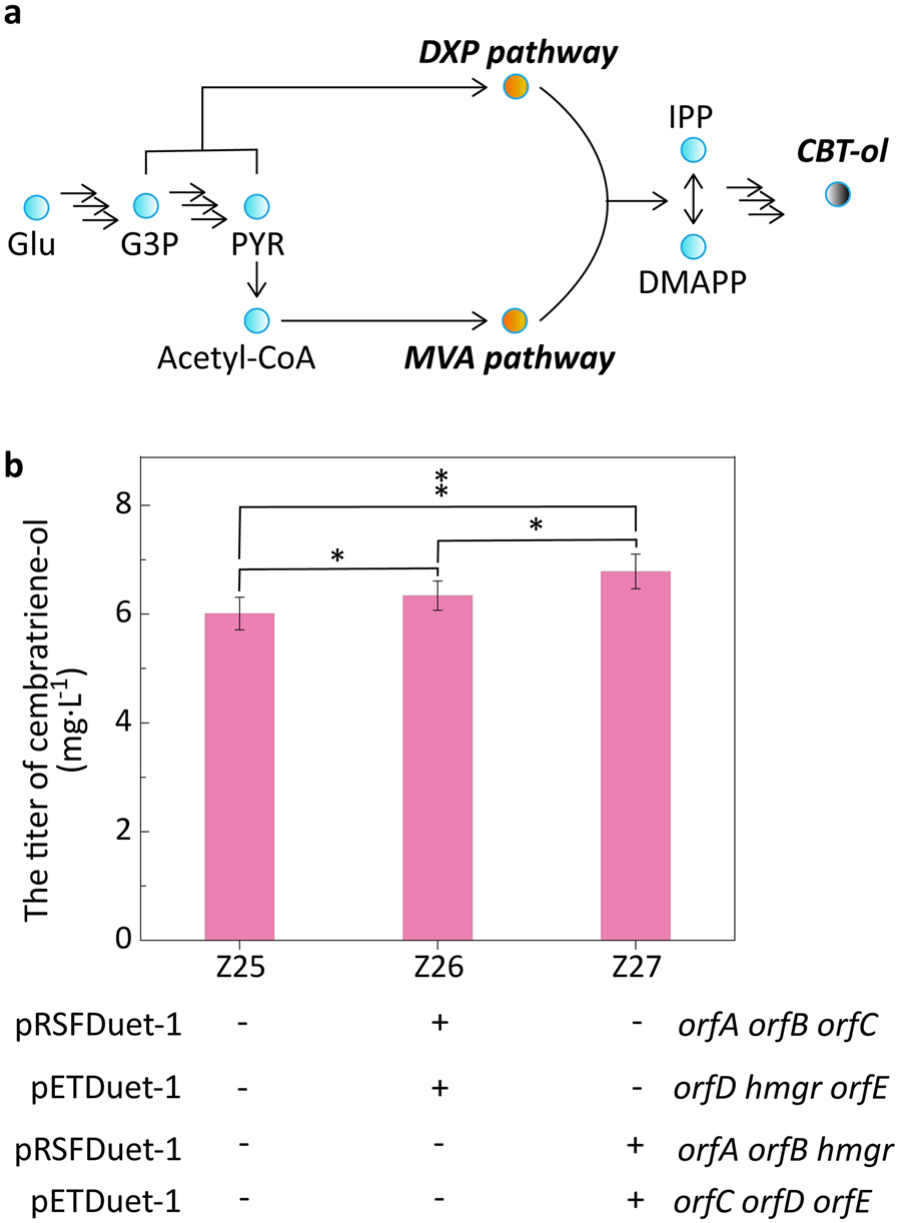
Introduction of the heterologous MVA pathway. **a** Schematic diagram of introduction of the heterologous MVA pathway in *E*. *coli*. **b** Effects of introduction of the heterologous MVA pathway on the production of cembratriene-ol at 24 h. Student’s *t*-test was used to statistically analyze data, and statistical significance was set at *p* value < 0.05. Asterisks indicate significant differences compared to *E*. *coli* Z25 (**, *p* < 0.05; *, *p* > 0.05). *p* < 0.05 was considered statistically significant

*E*. *coli* only has the natural DXP pathway, which includes 7 enzymatic steps with an adenosine triphosphate (ATP) and a reducing agent (NADPH) [1]. Intermediate enzymes and their genes from DXP pathway have been identified, and terpenoid units are synthesized from DXP starting from the condensation of glyceraldehyde 3-phosphate and pyruvate in this pathway [43, 44]. Other alternative routes (e.g., from pentose) along with original DXP pathway can be utilized to improve several terpenoids production [1]. MVA pathway commonly found in archaea and eukaryotes is another known biosynthetic pathway of terpenoids [1, 45]. MVA pathway starting from condensation of 2 acetyl-CoA includes 6 enzymatic steps with 3 ATPs and 2 NADPHs [44]. The heterologous MVA pathway was commonly introduced to improve production of terpenoids in *E*. *coli* [1]. In this study, 6 genes (*orfA*, *orfB*, *orfC*, *orfD*, *hmgr*, and *orfE*) encoding enzymes from MVA pathway of *Streptomyces* were synthesized after codon optimization to introduce a heterologous MVA pathway in *E*. *coli* Z25, including acetyl-CoA carboxylase, acetoacetyl-CoA synthase, hydroxymethylglutaryl-CoA synthase, hydroxymethylglutaryl-CoA reductase, mevalonate kinase, and mevalonate diphosphate decarboxylase (Fig. 4a). Genes *orfA*, *orfB*, and *orfC* were overexpressed with pRSFDuet-1 and genes *orfD*, *hmgr*, and *orfE* were overexpressed with pETDuet-1 to construct *E*. *coli* Z26 (Additional file 1: Fig. S13a). Genes *orfA*, *orfB*, and *hmgr* were overexpressed with pRSFDuet-1 and genes *orfC*, *orfD*, and *orfE* were overexpressed with pETDuet-1 to construct *E*. *coli* Z27 (Additional file 1: Fig. S13a). Production of cembratriene-ol was enhanced by introduction of the heterologous MVA pathway from *Streptomyces*. The titer of cembratriene-ol in *E*. *coli* Z27 was significantly increased by 1.13-folds that of *E*. *coli* Z25 (*p* value < 0.05) at 24 h (Fig. 4b). However, the titer of cembratriene-ol in *E*. *coli* Z27 was not significantly improved compared with that of *E*. *coli* Z26. Overexpression of key enzyme genes using plasmids with high copy numbers were also preferred for production of terpenoids [1, 46]. The conversion yields of glucose to cembratriene-ol in *E*. *coli* Z26 and *E*. *coli* Z27 were lower than that of *E*. *coli* Z25, which were consistent with glucose consumption yields of these strains (Additional file 1: Fig. S13b). Meanwhile, it was also found that with the extension of fermentation time, the titers of cembratriene-ol in *E*. *coli* Z26 and *E*. *coli* Z27 were not significantly improved compared with that of *E*. *coli* Z25 (data not shown). It was presumed that the introduction of the heterologous MVA pathway from *Streptomyces* might cause a metabolic imbalance in the late fermentation stage. Glucose was not gradually converted into cembratriene-ol in *E*. *coli* Z26 and *E*. *coli* Z27. However, cell growth of *E*. *coli* Z26 and *E*. *coli* Z27 was inhibited compared with that of *E*. *coli* Z25 (Additional file 1: Fig. S13c/d). More byproducts were formed in *E*. *coli* Z26 and *E*. *coli* Z27 than *E*. *coli* Z25. For example, the concentrations of lactic acid in *E*. *coli* Z26 and *E*. *coli* Z27 reached 90 and 69 mg g^− 1^ (DCW), which were higher than that in *E*. *coli* Z25. Meanwhile, the acetic acid concentrations in *E*. *coli* Z26 and *E*. *coli* Z27 reached 3.15 and 5.15 g g^− 1^ (DCW), which were higher than that (2.89 g g^− 1^ (DCW)) in *E*. *coli* Z25 (Additional file 1: Fig. S14).

### Enhancing the production of cembratriene-ol via overexpressing *ispD* and *ispF* and fermentation scale-up in 15-L fermenter

**Fig. 5:**
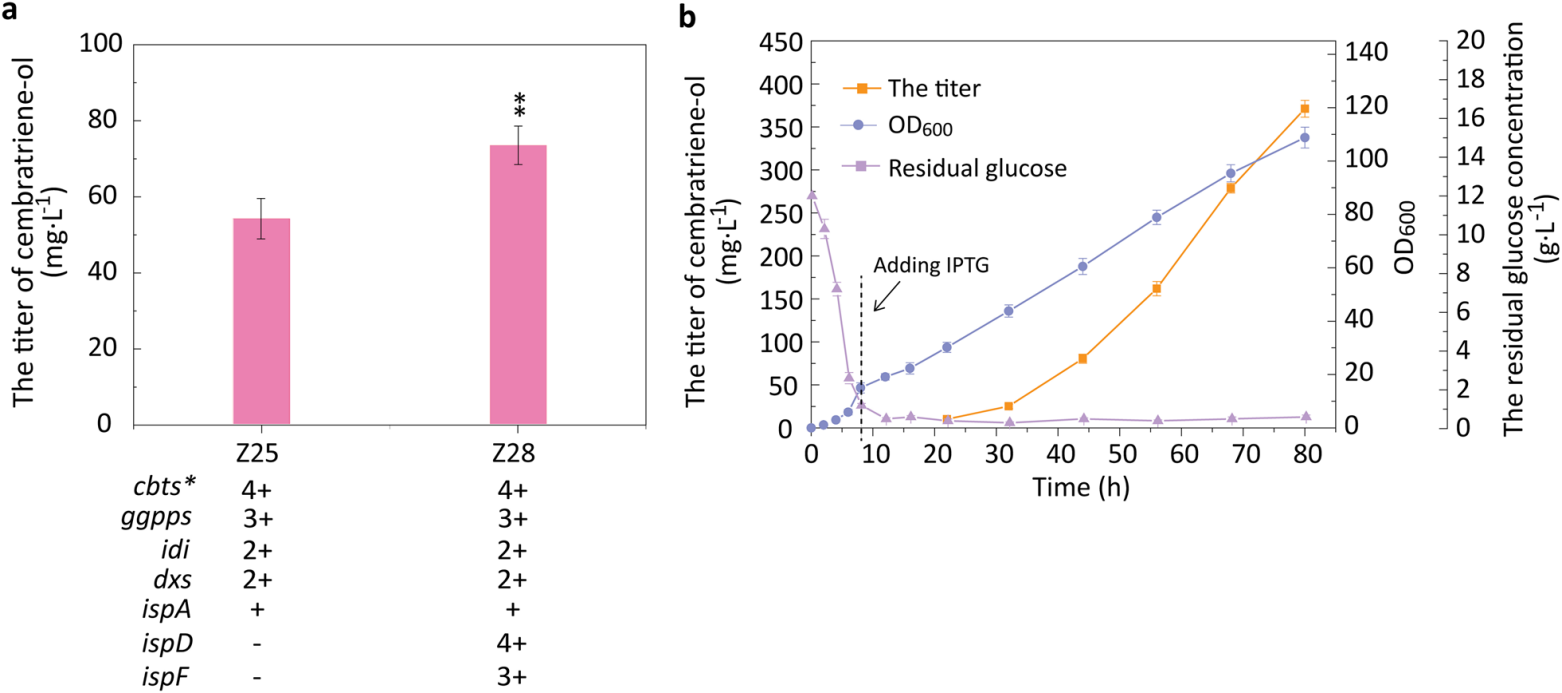
Enhancing the production of cembratriene-ol via overexpressing *ispD* and *ispF* and fermentation scale-up in 15-L fermenter. a, Enhancing the production of cembratriene-ol via overexpressing *ispD* and *ispF*. −, no gene overexpression; +, gene overexpression using pACYCDuet-1; 2+, gene overexpression using pCDFDuet-1; 3+, gene overexpression using pETDuet-1; 4+, gene overexpression using pRSFDuet-1. Student’s *t*-test was used to statistically analyze data, and statistical significance was set at *p* value < 0.05. Asterisks indicate significant differences compared to *E*. *coli* Z25 (**, *p* < 0.05). *p* < 0.05 was considered statistically significant. b, Fermention scale-up in 15-L fermentor

It was found that the titers of cembratriene-ol in *E*. *coli* Z26 and *E*. *coli* Z27 heterologously overexpressing MVA pathway from *Streptomyces* were not significantly improved compared with that of the control (*E*. *coli* Z25). The enzyme encoded by *dxs* can condensate glyceraldehyde-3-phosphate with pyruvate to produce deoxy-d-xylulose in the DXP pathway, and deoxy-d-xylulose is converted to the C5 isoprene subunits (IPP and DMAPP) by a series of additional isoprenoid enzymes encoded by *dxr*, *ispD*, *ispE*, *ispF*, *ispG*, and *ispH* in subsequent sequential reactions [33]. Enzymatic bottlenecks genes (*dxs*, *idi*, *ispD*, and *ispF*) reported were overexpressed to increase the flux through the upstream DXP pathway [33, 47]. Therefore, key genes *ispD* and *ispF* from the DXP pathway were additionally overexpressed to enhance synthesis of intermediate metabolites to improve the accumulation of cembratriene-ol in this study. The genes *ispD* and *ispF* were overexpressed using pRSFDuet-1 and pETDuet-1 with high copy numbers in *E. coli* Z25 to construct the recombinant strain *E. coli* Z28 (Additional file 1: Table S1). The cembratriene-ol titer in *E*. *coli* Z28 reached 83.91 mg L^− 1^ at 72 h, which was increased by 1.56-folds that of *E*. *coli* Z25 (Fig. 5a).

Through fermentation optimization in the 250 mL shaking flask, it was verified that addition of glucose and organic nitrogen source (yeast extract) was conducive to the production of cembratriene-ol and cell growth. However, the concentration of added glucose in the shaking flask was low and not used for high-density fermentation in a 15-L fermenter. In this study, a fed-batch culture with DO-stat was adopted to achieve high-density fermentation in a 15-L fermenter. The feeding medium was fed at a dissolved oxygen (DO) concentration of 45%. Among feeding media, the carbon source was glucose, and the nitrogen source was mainly provided by adding NH_3_ H_2_O (inorganic nitrogen source). In order to promote cell growth, a small amount of yeast extract and peptone was added in high concentration glucose fed to supplement a certain amount of organic nitrogen source. Feeding medium included 500 g L^− 1^ glucose, 7.33 g L^− 1^ MgSO_4_, 4.0 g L^− 1^ yeast extract, and 4.0 g L^− 1^ tryptone. When the cell density OD_600_ reached 15 at 8 h, IPTG was added to induce expression of key enzyme genes (e.g. *dxs*, *idi*, *ispA*, *ispD*, *ispF*, *ggpps*, and cbts*), which could be used to efficiently synthesize cembratriene-ol in *E*. *coli* Z28. The titers of cembratriene-ol in 15-L fermenter reached 371.2 mg L^− 1^ at 80 h, which was significantly enhanced compared with that of the initially constructed *E*. *coli* Z14 (Fig. 5b). The highest OD_600_ reached 102.3 at 80 h. The residual glucose concentrations were below 0.5 g L^− 1^ (Fig. 5b). Carbon substrate feeding speed was connected to the DO level. When *E*. *coli* cells were actively growing with sufficient glucose, they required a lot of oxygen and the DO level would become very low. Once glucose is depleted, the DO level started to rise. Meanwhile, the titer of cembratriene-ol in the recombinant *E*. *coli* overexpressing *dxs*, *idi*, and *ggpps* using different ribosomal binding sites (RBS) combinatorics reached 78.9 mg L^− 1^ at 120 h in a 50-L fermenter, and the dry cell weight reached 15.3 g L^− 1^ [12].

## Conclusion

The tobacco leaf–derived cembratriene-ol was successfully synthsized in recombinant *E*. *coli*. The systematic regulation of *ggpps*, cbts*, *dxs*, and *idi* expression levels could promote the production of cembratriene-ol. The overexpression of the farnesyl diphosphate synthase gene *ispA*, *ispD*, and *ispF* was significantly for enhancement of the cembratriene-ol titer. The cembratriene-ol titer in recombinant *E*. *coli* could be importantly improved by fermentation optimisation and introduction of the heterologous MVA pathway from *Streptomyces*. The cembratriene-ol titer in the 15-L fermenter was the highest titer reported. Systematic optimization has great potential in facilitating high-level production of cembratriene-ol production in *E. coli*.

## Materials and methods

### Strains and plasmids

The strains constructed and used are listed in Additional file 1: Table S1. *E. coli* JM109 was used for cloning, and *E. coli* BL21(DE3) was used as the initial strain to produce cembratriene-ol. The plasmids used and constructed are listed in Additional file 1: Table S2. The primers used for gene cloning and plasmid construction are listed in Additional file 1: Table S3.

### Chemical reagents and kits

The antibiotics used in this study included ampicillin (Amp), kanamycin (Kan), streptomycin (Sm), and chloramphenicol (Cm). The standard of cembratriene-ol was purchased from Boc Sciences (Shiriey, New York, United States). Yeast extract and tryptone were purchased from Thermo Fisher Scientific Ltd. (Waltham, Massachusetts, USA), glucose, isopropyl-β-d-thiogalactose (IPTG), and L-arabinose were purchased from Sangon Biotech (Shanghai) Co., Ltd.; *n*-hexane and ethyl acetate were purchased from Shanghai Aladdin Biochemical Technology Co., Ltd.; PCR enzymes (e.g. PrimeSTAR HS DNA polymerase), ligase solution I, and restriction endonucleases needed for molecular biology experiments were purchased from TaKaRa (Dalian, China). The kits for plasmid extraction, agarose gel DNA extraction, and DNA fragment purification were purchased from Axygen Scientific, Inc. (Union, California, USA).

### Media and culture conditions

Lysogeny broth (LB) medium (1 L) included 10 g NaCl, 10 g tryptone, and 5 g yeast extract. Solid LB medium was supplemented with 2% (w/v) agar powder. 5×M9 medium (200 mL) includes 12.8 g Na_2_PO_4_·7H_2_O, 3.0 g KH_2_PO_4_, 0.5 g NaCl, 1.0 g NH_4_Cl. Initial fermentation medium (1 L) includes 200 mL 5×M9 medium, 2 mL 1 M MgSO_4_ solution, 0.1 mL 1 M CaCl_2_ solution, 20 mL 20% (w/v) glucose solution, 1 mL 1 g L^− 1^ thiamine solution, 1 mL 1 g L^− 1^ biotin solution. A single colony from solid LB medium was transferred into 50 mL liquid LB medium in a 250 mL flask, which was cultured at 37 °C and 200 rpm for 10 h. The fermentation conditions for producing cembratriene-ol in 250 mL flasks (75 mL fermentation media) were a 1% (v/v) inoculum, 30 °C, 200 rpm, and 150 µM IPTG (final concentration) and lowering the fermentation temperature to 22 °C until OD_600_ = 0.6. Cells were collected at 5000 ×*g* at 4 °C for 10 min. The fermentation medium in 15-L fermenter included 12.0 g L^− 1^ glucose, 13.5 g L^− 1^ KH_2_PO_4_, 4.0 g L^− 1^ (NH_4_)_2_PO_4_, 1.7 g L^− 1^ citric acid, 1.68 g L^− 1^ MgSO_4_, and 5 mL L^− 1^ trace element solution. Feeding medium included 500 g L^− 1^ glucose, 7.33 g L^− 1^ MgSO_4_, 4.0 g L^− 1^ yeast extract, and 4.0 g L^− 1^ tryptone. Trace element solution included 100 mg L^− 1^ FeSO_4_ 7H_2_O, 52.5 mg L^− 1^ ZnSO_4_ 7H_2_O, 30 mg L^− 1^ CuSO_4_ 2H_2_O, 5 mg L^− 1^ MnSO_4_ 4H_2_O, 2.3 Borax, 20 mg L^− 1^ CaCl_2_, and 1 mg L^− 1^ (NH_4_)_2_MoO_4_. Among feeding medium, the carbon source was glucose, and the nitrogen source was mainly provided by adding NH_3_ H_2_O (inorganic nitrogen source). In order to promote cell growth, a small amount of yeast extract (4.0 g L^− 1^) and peptone (4.0 g L^− 1^) was added in 500 g L^− 1^ glucose fed to supplement a certain amount of organic nitrogen source. Strains were first cultured in 200 mL LB medium (2 L shaking flask), 1 L of which was transformed into a 15-L fermenter when the OD_600_ in the shaking flask reached 0.8. The 15-L fermenter included 7 L of fermentation medium, and the pH was controlled at 7.0 by adding NH_3_ H_2_O. The initial fermentation conditions were as follows: 37 °C, 200 rpm (initial speed), dissolved oxygen (DO) = 50%, and 1.0 MPa. 150 µM IPTG (final concentration) was added when OD_600_ reached 15, and the culture temperature was changed to 30 °C. The feeding medium was fed at a DO concentration of 45%.

### Construction of recombinant plasmids and cembratriene-ol production strains

*dxs* gene was amplified from the *E. coli* BL21 (DE3) genome using primers *dxs*-FW and *dxs*-RS, which were digested with restriction endonucleases *Nde* I and *Xho* I. It was then ligated with pCDFDuet-1 digested with the same restriction enzymes to construct the recombinant plasmid pCDFDuet-1-*dxs*. Isopentenyl diphosphate isomerase (GenBank No. AAC32208.1) gene *idi* from *H*. *lacustris*, geranylgeranyl pyrophosphate synthetase (CrtE, GenBank no. ADD79325.1) gene *ggpps* from *P*. *ananatis* LMG 20,103, and the cembratriene-ol synthase CBTS (GenBank no. AAS46038.1) genes *cbts* and cbts* without the plastid transit peptide (MSQSISPLICSHFAKFQSNIWRCNTSQLRVIHSSYASFGGRRKERVRRMNRA) from *N*. *tabacum*, acetyl-CoA carboxylase gene *orfA* (NCBI Reference Sequence: WP_142233022.1), acetoacetyl-CoA synthase gene *orfB* (GenBank no. EGE45775.1), hydroxymethylglutaryl-CoA synthase gene *orfC* (NCBI Reference Sequence: WP_150173950.1), hydroxymethylglutaryl-CoA reductase gene *orfD* (NCBI Reference Sequence: WP_150173948.1), mevalonate kinase gene *hmgr* (GenBank no. BAB07790.1), and mevalonate diphosphate decarboxylase gene *orfF* (GenBank no. BAD86801.1) from *Streptomyces* were synthesised by Suzhou GENEWIZ Biological Technology Co., Ltd. (Suzhou, China) after codon optimization in *E*. *coli* (Supplemental materials). Gene *idi* was ligated with pCDFDuet-1 to construct pCDFDuet-1-*idi* after digestion using *Nco* I and *Eco*R I. After digestion via *Nde* I and *Xho* I, *idi* was ligated with pCDFDuet-1-*dxs* to construct recombinant plasmid pCDFDuet-1-*dxs-idi*. After digestion with *Nde* I and *Xho* I, *ggpps* was ligated with plasmid pETDuet-1 and pRSFDuet-1 to obtain the recombinant plasmids pETDuet-1-*ggpps* and pRSFDuet-1-*ggpps*. The *cbts* and cbts* genes were digested with *Nco* I and *Eco*R I and ligated to plasmids pETDuet-1-*ggpps* and pRSFDuet-1-*ggpps* to obtain recombinant plasmids pETDuet-1-*ggpps-cbts*, pETDuet-1-ggpps-cbts*, and pRSFDuet-1-ggpps-cbts*, respectively. The digested cbts* gene was ligated with pETDuet-1 and pRSFDuet-1 to obtain the recombinant plasmids pETDuet-1-cbts* and pRSFDuet-1-cbts*. The digested gene *dxs* was ligated with the plasmid pACYCDuet-1 to obtain the recombinant plasmid pACYCDuet-1-*dxs*. Digested *idi* was ligated with plasmids pCDFDuet-1, pACYCDuet-1, and pACYCDuet-1-*dxs* to obtain the recombinant plasmids pCDFDuet-1-*idi*, pACYCDuet-1-*idi*, and pACYCDuet-1-*dxs-idi*. The *ispA* gene was cloned from *E. coli* BL21 (DE3) genome using primers *ispA_Ecoli*_FW and *ispA_Ecoli*_RS and ligated with pACYCDuet-1 to construct the recombinant plasmid pACYCDuet-1-*ispA*. Recombinant plasmids were transformed into *E. coli* JM109 or *E. coli* BL21 (DE3) to obtain recombinant *E*. *coli* Z1 − Z28.

### Detection of cembratriene-ol

After fermentation, 30 mL of fermentation broth with cells was collected from each flask. All flask fermentations were performed in triplicate. Cells in each flask were lysed using a SCIENTZ-IID Ultrasonic Cell Crusher from Ningbo Scientz Biotechnology Co., Ltd. (Ningbo, China). Equal volumes of the extractant (*n*-hexane:ethyl acetate = 1:1) were added to the mixtures, lysed, mixed for 30 min, and centrifuged at 5000×*g* and 4 °C for 10 min. The supernatant was collected and dried at 30 °C using a RE100-Pro rotary evaporator (Scilogex, New Hampshire, USA). To reconstitute the dried samples, 3 mL of ethyl acetate was used, and reconstitution was determined using GC-MS. The TraceGOLD TG-5MS GC column (30 m × 0.25 mm × 0.25 µm) of Thermo Fisher Scientific Co., Ltd. (Waltham, USA) was used to determine cembratriene-ol. The column temperature was 100 °C and its hold time was 0 min; it was increased to 250 °C at a rate of 10 °C min^– 1^ and its hold time was 5 min. MS data of samples were recorded at 70 eV (EI) and *m*/*z* (rel. intensity in %) as total ion current. Full scan mode (*m*/*z* 35–350) was used to collect the data. The productivity of cembratriene-ol was calculated over the whole culture. The conversion yield of glucose to cembratriene-ol was calculated by dividing the cembratriene-ol titer (mg L^– 1^) by the glucose consumption concentration (g L^– 1^). Three parallel experiments were independently performed, and data are reported as mean ± standard deviation (SD).

### Determination of the cell density (OD_600_) and the specific growth rate

Recombinant *E*. *coli* culture broth (1 mL) was used to determine the absorbance at 600 nm (OD_600_). The specific growth rate plots were drawn from OD measurements using the software OriginPro 8.5.1. The data processing steps are as follows: (a) Fitting of time and OD to obtain data 1; (b) Differential of OD to time to obtain data 2; (c) Data 2 were divided by data 1 to obtain specific growth rates (µ), which included 100 data and the total fermentation time was evenly divided into 100 equal parts. The obtained specific growth rates (µ) including 100 data and time divided into 100 equal parts were used to draw the specific growth rate plots using the software OriginPro 8.5.1. Three parallel experiments were independently performed, and data are reported as mean ± standard deviation (SD).

### Determination of residual glucose

Fermentation broth (1 mL) was collected and centrifuged at 12,000×*g* at 4 °C for 3 min. The supernatant was collected, and the glucose concentration was determined using a residual sugar analyser, SGD-III (Shandong Academy of Sciences, Jinan, China). Three parallel experiments were independently performed, and data are reported as mean ± standard deviation (SD).

## Supplementary Information

**Additional file 1.** Additional experimental section. **Table S1.** Strains used and constructed in this study. **Table S2.** Plasmids used and constructed in this study. **Table S3.** Primers used in this study. Gene Sequence of *cbts* optimized. Gene Sequence of *ggpps* optimized. Gene sequence of *idi* optimized. Gene sequence of *orfA* optimized. Gene sequence of *orfB* optimized. Gene sequence of *orfC* optimized. Gene sequence of *orfD* optimized. Gene sequence of hmgr optimized. Gene sequence of *orfE* optimized. Gene sequence of *ispD*. Gene sequence of *ispF*. **Figure S1.** Efficient synthesis of cembratriene-ol via systematic optimization in this study. **Figure S2.** Gas chromatographic (GC) profile and the associated mass peaks of cembratriene-ol. **Figure S3.** Effects of expression of cembratriene-ol synthase gene *cbts* on growth of recombinant *E. coli*. **Figure S4.** The residual glucose concentrations of different strains. **Figure S5.** Effects of expression of the cembratriene-ol synthase truncated variant gene cbts* on growth of recombinant *E. coli*. **Figure S6.** SDS-PAGE of CBTS and CBTS* expressed in *E. coli*. **Figure S7.** Effects of overexpression of *dxs* and *idi* on growth of recombinant *E. coli* **Figure S8.** Effects of overexpressing key genes on growth of recombinant *E. coli*. **Figure S9.** Effects of systematic regulating the expression level of key genes on growth of recombinant *E. coli* **Figure S10.** The copy numbers of different plasmids at the cultivation conditions in this study. **Figure S11.** Effects of overexpression of gene *ispA* on growth of recombinant *E. coli*. **Figure S12.** Effects of fermentation optimization on growth of recombinant *E. coli*. **Figure S13.** Effects of introduction of the heterologous MVA pathway on the glucose utilization, conversion yield, and growth of recombinant *E. coli*. **Figure S14.** The concentrations of lactic acid and acetic acid in *E. coli* Z26 and *E. coli* Z27.

## Data Availability

All data for this study are included in this published article and its additional file.
