# Supplementary material for: Efficient production of cembratriene-ol in Escherichia coli via systematic optimization
Source: Microb Cell Fact. 2023 Jan 24;22:17. doi: 10.1186/s12934-023-02022-4 (PMC9872381; doi:10.1186/s12934-023-02022-4)
Supplement: Supplementary file 1 — Additional file 1. Additional experimental section. Table S1. Strains used and constructed in this study. Table S2. Plasmids used and constructed in this study. Table S3. Primers used in this study. Gene Sequence of cbts optimized. Gene Sequence of ggpps optimized. Gene sequence of idi optimized. Gene sequence of orfA optimized. Gene sequence of orfB optimized. Gene sequence of orfC optimized. Gene sequence of orfD optimized. Gene sequence of hmgr optimized. Gene sequence of orfE optimized. Gene sequence of ispD. Gene sequence of ispF. Figure S1. Efficient synthesis of cembratriene-ol via systematic optimization in this study. Figure S2. Gas chromatographic (GC) profile and the associated mass peaks of cembratriene-ol. Figure S3. Effects of expression of cembratriene-ol synthase gene cbts on growth of recombinant E. coli. Figure S4. The residual glucose concentrations of different strains. Figure S5. Effects of expression of the cembratriene-ol synthase truncated variant gene cbts* on growth of recombinant E. coli. Figure S6. SDS-PAGE of CBTS and CBTS* expressed in E. coli. Figure S7. Effects of overexpression of dxs and idi on growth of recombinant E. coli Figure S8. Effects of overexpressing key genes on growth of recombinant E. coli. Figure S9. Effects of systematic regulating the expression level of key genes on growth of recombinant E. coli Figure S10. The copy numbers of different plasmids at the cultivation conditions in this study. Figure S11. Effects of overexpression of gene ispA on growth of recombinant E. coli. Figure S12. Effects of fermentation optimization on growth of recombinant E. coli. Figure S13. Effects of introduction of the heterologous MVA pathway on the glucose utilization, conversion yield, and growth of recombinant E. coli. Figure S14. The concentrations of lactic acid and acetic acid in E. coli Z26 and E. coli Z27. [file 12934_2023_2022_MOESM1_ESM.docx]

**Efficient production of cembratriene-ol in *Escherichia coli* via systematic optimization**

Haiquan Yang^1^, Kunjie Zhang^1^, Wei Shen^2^, Lei Chen^1^, Yuanyuan Xia^2^, Wei Zou^3^, Yu Cao^2^, Xianzhong Chen^2,*^

^1^ The Key Laboratory of Carbohydrate Chemistry and Biotechnology, Ministry of Education, School of Biotechnology, Jiangnan University, Wuxi 214122, China

^2^ The Key Laboratory of Industrial Biotechnology, Ministry of Education, School of Biotechnology, Jiangnan University, Wuxi 214122, China

^3^ College of Bioengineering, Sichuan University of Science & Engineering, Yibin, Sichuan 644000, China

*Corresponding author. E-mail: xzchen@jiangnan.edu.cn (X.C.). Fax: +86 0510 85918122.

**Table S1** Strains used and constructed in this study

| Strains | Genotype and related characteristics | Sources |
| --- | --- | --- |
| *E. coli* JM109 | Cloning host | CTCC |
| *E. coli* BL21(DE3) | F^−^ *ompThsdSB*(*rBmB*) *gal dcm* (DE3) | Invitrogen |
| *E. coli* Z0 | *E. coli* BL21(DE3) contains plasmid pETDuet-1 | This study |
| *E. coli* Z1 | *E. coli* JM109 contains plasmid pCDFDuet-1-*dxs* | This study |
| *E. coli* Z2 | *E. coli* JM109 contains plasmid pCDFDuet-1*-dxs-idi* | This study |
| *E. coli* Z3 | *E. coli* JM109 contains plasmid pETDuet-1-*ggpps* | This study |
| *E. coli* Z4 | *E. coli* JM109 contains plasmid pRSFDuet-1-*ggpps* | This study |
| *E. coli* Z5 | *E. coli* JM109 contains plasmid pETDuet-1*-ggpps-cbts* | This study |
| *E. coli* Z6 | *E. coli* JM109 contains plasmid pETDuet-1-*ggpps-cbts** | This study |
| *E. coli* Z7 | *E. coli* JM109 contains plasmid pRSFDuet-1-*ggpps-cbts** | This study |
| *E. coli* Z8 | *E. coli* JM109 contains plasmid pETDuet-1-*cbts** | This study |
| *E. coli* Z9 | *E. coli* JM109 contains plasmid pRSFDuet-1-*cbts** | This study |
| *E. coli* Z10 | *E. coli* JM109 contains plasmid pACYCDuet-1-*dxs* | This study |
| *E. coli* Z11 | *E. coli* JM109 contains plasmid pACYCDuet-1-*idi* | This study |
| *E. coli* Z12 | *E. coli* JM109 contains plasmid pCDFDuet-1*-idi* | This study |
| *E. coli* Z13 | *E. coli* JM109 contains plasmid pACYCDuet-1-*dxs-idi* | This study |
| *E. coli* Z14 | *E. coli* BL21(DE3) contains plasmid pETDuet-1-*ggpps-cbts* | This study |
| *E. coli* Z15 | *E. coli* BL21(DE3) contains plasmid pETDuet-1-*ggpps-cbts** | This study |
| *E. coli* Z16 | *E. coli* Z15 contains plasmid pACYCDuet-1-*dxs-idi* | This study |
| *E. coli* Z17 | *E. coli* Z15 contains plasmid pCDFDuet-1-*dxs-idi* | This study |
| *E. coli* Z18 | *E. coli* BL21(DE3) contains plasmids pCDFDuet-1-*dxs-idi* and pRSFDuet-1-*ggpps-cbts** | This study |
| *E. coli* Z19 | *E. coli* BL21(DE3) contains plasmids pCDFDuet-1-*dxs-idi*, pETDuet-1-*cbts** and pRSFDuet-1-*ggpps* | This study |
| *E. coli* Z20 | *E. coli* BL21(DE3) contains plasmids pCDFDuet-1-*dxs-idi*, pRSFDuet-1-*cbts** and pETDuet-1-*ggpps* | This study |
| *E. coli* Z21 | *E. coli* BL21(DE3) contains plasmids pETDuet-1-*ggpps-cbts**, pACYCDuet-1-*dxs*, and pCDFDuet-1*-idi* | This study |
| *E. coli* Z22 | *E. coli* BL21(DE3) contains plasmids pETDuet-1-*ggpps-cbts**, pCDFDuet-1-*dxs* and pACYCDuet-1-*idi* | This study |
| *E. coli* Z23 | *E. coli* JM109 contains plasmid pACYCDuet-1-*ispA* | This study |
| *E. coli* Z24 | *E. coli* Z20 contains plasmid pACYCDuet-1 | This study |
| *E. coli* Z25 | *E. coli* Z20 contains plasmid pACYCDuet-1*-ispA* | This study |
| *E. coli* Z26 | *E. coli* BL21(DE3) contains plasmids pACYCDuet-1*-ispA*, pRSFDuet-1-*cbts**-*orfA*-*orfB*-*orfC* pETDuet-1*-orfD-hmgr-orfE*-*ggpps*, and pCDFDuet-1-*idi*-*dxs* | This study |
| *E. coli* Z27 | *E. coli* BL21(DE3) contains plasmids pACYCDuet-1*-ispA*, pRSFDuet-1-*cbts**-*orfA*-*orfB*-*hmgr*, pETDuet-1*-orfC- orfD-orfE*-*ggpps*, and pCDFDuet-1-*idi*-*dxs* | This study |
| *E. coli* Z28 | *E. coli* BL21(DE3) contains plasmids pACYCDuet-1*-ispA*, pRSFDuet-1-*cbts**-*ispD*, pETDuet-1*-ispF*-*ggpps*, and pCDFDuet-1-*idi*-*dxs* | This study |

**Table S2** Plasmids used and constructed in this study

| Plasmids | Genotype and related characteristics | | Sources |
| --- | --- | --- | --- |
| pCDFDuet-1 | Double T7 promoter, two multiple cloning sites, sm^R^ | Novagen | |
| pETDuet-1 | Double T7 promoter, two multiple cloning sites, amp^R^ | Novagen | |
| pRSFDuet-1 | Double T7 promoter, two multiple cloning sites, kan^R^ | Novagen | |
| pACYCDuet-1 | Double T7 promoter, two multiple cloning sites, cm^R^ | Novagen | |
| pCDFDuet-1*-dxs* | pCDFDuet-1 contains *dxs* gene | This study | |
| pCDFDuet-1-*dxs-idi* | pCDFDuet-1 contains *dxs* and *idi* gene | This study | |
| pETDuet-1-*ggpps* | pETDuet-1 contains *ggpps* gene | This study | |
| pETDuet-1*-cbts** | pETDuet-1 contains *cbts** gene | This study | |
| pETDuet-1-*ggpps-cbts* | pETDuet-1 contains *ggpps* and *cbts* gene | This study | |
| pETDuet-1-*ggpps-cbts** | pETDuet-1 contains *ggpps* and *cbts** gene | This study | |
| pRSFDuet-1-*ggpps* | pRSFDuet-1 contains *ggpps* gene | This study | |
| pRSFDuet-1-*ggpps- cbts** | pRSFDuet-1 contains *ggpps* and *cbts** gene | This study | |
| pRSFDuet-1-*cbts** | pRSFDuet-1 contains *cbts** gene | This study | |
| pACYCDuet-1-*dxs* | pACYCDuet-1 contains *dxs* gene | This study | |
| pACYCDuet-1-*idi* | pACYCDuet-1 contains *idi* gene | This study | |
| pACYCDuet-1-*dxs-idi* | pACYCDuet-1 contains *dxs* and *idi* gene | This study | |
| pCDFDuet-1-*idi* | pCDFDuet-1 contains *idi* gene | This study | |
| pACYCDuet-1-*ispA* | pACYCDuet-1 contains *ispA* gene | This study | |
| pRSFDuet-1-*cbts**-*ispD* | pRSFDuet-1 contains *cbts** and *ispD* genes | This study | |
| pETDuet-1*-ispF*-*ggpps* | pETDuet-1 contains *ispF* and *ggpps* genes | This study | |

**Table S3** Primers used in this study

| Oligonucleotide primers | Sequences (5’→3’) |
| --- | --- |
| *dxs*-FW^a^ | CC*CATATG*ATGAGTTTTGATATTGCCAA |
| *dxs*-RS^a^ | CC*CTCGAG*TTATGCCAGCCAGGCCTTGA |
| *ispA_Ecoli*_FW^b^ | CATG*CCATGG*GCATGGACTTTCCGCAGCAACTC |
| *ispA_Ecoli*_RS^b^ | G*GAATTC*TTATTTATTACGCTGGATGAT |
| *ispD*-FW^c^ | GA*AGATCT*ATGGCAACCACTCATTTGGA |
| *ispD*-RS^c^ | GG*GGTACC*TTATGTATTCTCCTGATGGA |
| *ispF*-FW^d^ | CG*GGATCC*ATGCGAATTGGACACGGTTT |
| *ispF*-RS^d^ | G*GAATTC*TCATTTTGTTGCCTTAATGA |
| *pyrF*-FW^e^ | CG*GGATCC*ATGACGTTAACTGCTTCATC |
| *pyrF*-RS^e^ | CCC*AAGCTT*TTATGCACTCCGCTGTAAAG |

^a^Italic, restriction enzyme sites (*Nde* Ι and *Xho* Ι). ^b^Italic, restriction enzyme sites (*Nco* Ι and *Eco*R Ι). ^c^Italic, restriction enzyme sites (*Bgl* IΙ and *Kpn* Ι). ^d^Italic, restriction enzyme sites (*BamH* Ι and *Eco*R Ι). ^e^Italic, restriction enzyme sites (*BamH* Ι and *Hind* IIΙ).

**Gene sequence of *cbts* optimized**

ATGAGTCAGAGCATTAGCCCGCTCATTTGCAGCCACTTCGCCAAGTTCCAGAGTAACATCTGGCGCTGCAACACCAGCCAACTCCGTGTTATCCACAGCAGCTACGCGAGCTTCGGTGGTCGCCGTAAAGAACGTGTTCGCCGTATGAATCGCGCGATGGATCTCAGCAGCAGCAGTCGCCATCTGGCCGATTTTCCGAGTACGATCTGGGGTGACCACTTTCTGAGTTATAATAGTGAGATCACGGAGATCACCACCCAAGAAAAGAATGAGCACGAGATGCTCAAGGAGATTGTGCGCAAGATGCTGGTGGAAACCCCGGATAATAGCACGCAGAAGCTGGTGCTGATTGATACCATTCAGCGTCTGGGTCTGGCGTACCACTTCAACGATGAAATCGAAAACAGCATTCAAAACATTTTTAATCTGAGCCAGAACAGCGAGGATGACGACGAACACAATCTGTACGTTGCCGCGCTCCGTTTCCGTCTGGCGCGTCAACAAGGCTATTACATGAGCAGTGACGTTTTCAAGCAGTTCACGAACCACGACGGCAAGTTCAAGGAGAACCATACCAATGACGTTCAAGGTCTGCTGAGCCTCTACGAAGCGGCCCACATGCGCGTTCATGACGAGGAGATTCTGGAAGAGGCGCTGATCTTCACCACCACGCATCTGGAAAGCGTGATCCCGAATCTGAGCAACAGTCTGAAGGTGCAAGTTACGGAGGCGCTCAGCCATCCGATTCGTAAGGCCATTCCACGCGTTGGCGCGCGCAAATACATTCATATTTACGAAAATATCGGCACGCACAATGATCTGCTGCTGAAGTTCGCCAAGCTGGACTTCAACATGCTGCAGAAGCTGCACCGCAAGGAGCTGAACGAACTGACCAGCTGGTGGAAGGATCTGGATCGCGCCAACAAGTTTCCGTATGCGAAAGACCGTCTGGTGGAGGCGTACTTCTGGACGGTGGGCATCTATTTCGAACCGCAGTATAGCCGCAGCCGCAGTCTGGTGACGAAGGTGGTTAAGATGAACAGCATCATCGACGACACCTACGACGCCTACGCCACGTTCGATGAACTGGTGCTGTTCACCGACGCCATTCAGCGCTGGGACGAAGGTGCGATGGATCTGCTGCCGACGTATCTGCGCCCGATCTACCAAGGTCTGCTCGACGTGTTCAACGAGATGGAGGAGGTTCTGGCGAAGGAAGGTAAGGCCGACCACATTTACTACGCCAAGAAGGAGATGAAGAAGGTGGCCGAGGTTTACTTCAAGGAGGCGGAGTGGCTGAACGCGAACTACATCCCGAAGTGTGAAGAGTACATGAAGAATGGTCTGGTGAGCAGCACGGGTCCAATGTACGGCATCATCAGTCTGGTGGTGATGGAGGAGATCATCACGAAGGAAGCCTTCGAATGGCTGACCAATGAGCCACTGATTCTGCGCGCCGCGAGTACCATTTGTCGTCTGATGGACGATATGGCGGATCATGAGGTGGAACAACAGCGCGGCCACGTGGCCAGTTTCGTTGAATGCTACATGAAGGAGTACGGCGTGAGCAAACAAGAAACCTATGTGGAGATGCGCAAGAAGATTACCAACGCGTGGAAAGACATCAATAAGGAGCTGCTGCGCCCAACCGCGGTGCCGATGTTCATTCTGGAACGTAGTCTCAATTTCAGCCGTCTGGCCGATACGTTCCTCAAAGATGATGACGGCTACACCAATCCGAAGAGCAAGGTGAAGGATCTGATCGCGAGCCTCTTCGTTGAAAGTGTGGATTAA

**Gene sequence of *ggpps* optimized**

TACCCATTCATCCGTACCGCCCGCATGACCGTGTGCGCGAAGAAGCACGTGCATCTGACGCGTGATGCGGCGGAACAACTGCTGGCCGACATCGATCGCCGTCTGGATCAGCTGCTCCCAGTTGAAGGCGAGCGTGATGTTGTTGGTGCCGCCATGCGTGAAGGTGCCCTCGCGCCGGGCAAACGCATCCGCCCAATGCTGCTGCTGCTGACGGCGCGTGATCTGGGTTGCGCCGTTAGCCATGATGGTCTGCTGGACCTCGCGTGCGCCGTTGAAATGGTTCATGCGGCGAGTCTGATTCTGGACGATATGCCGTGCATGGATGATGCCAAACTGCGCCGTGGTCGTCCGACGATTCATAGCCACTACGGCGAACACGTTGCCATTCTGGCCGCGGTTGCGCTGCTGAGCAAAGCCTTTGGCGTTATTGCGGACGCGGATGGTCTCACCCCACTGGCGAAGAACCGCGCCGTTAGCGAACTGAGCAATGCGATCGGCATGCAAGGTCTGGTTCAAGGCCAGTTCAAGGATCTGAGCGAAGGCGATAAGCCACGCAGCGCGGAGGCGATTCTGATGACCAACCACTTCAAGACGAGTACGCTGTTTTGCGCCAGCATGCAGATGGCGAGCATCGTTGCCAACGCCAGCAGCGAAGCCCGCGACTGTCTGCACCGCTTCAGTCTGGATCTGGGTCAAGCCTTCCAGCTGCTCGATGATCTGACCGATGGCATGACCGACACCGGCAAGGATAGTAACCAAGATGCCGGTAAAAGCACGCTGGTGAATCTGCTGGGTCCACGCGCGGTTGAAGAACGTCTGCGCCAACATCTGCATCTCGCGAGCGAACATCTGAGCGCGGCGTGCCAGCATGGTCATGCGACCCAGCACTTCATCCAAGCGTGGTTCGACAAAAAGCTCGCGGCGGTTAGCTAA

**Gene sequence of *idi* optimized**

ATGCTGCGCAGTCTGCTCCGTGGTCTGACCCACATTCCACGCGTTAATAGCGCCCAGCAACCAAGTTGCGCCCATGCCCGTCTGCAGTTCAAACTGCGCAGCATGCAGATGACGCTGATGCAGCCGAGCATCAGCGCGAATCTGAGCCGCGCCGAAGATCGTACCGATCACATGCGTGGTGCGAGTACGTGGGCCGGTGGTCAGAGCCAAGATGAACTGATGCTGAAAGACGAGTGCATCCTCGTGGACGTTGAGGATAACATCACCGGTCACGCGAGCAAACTGGAATGCCACAAGTTTCTCCCGCACCAACCAGCCGGTCTGCTCCATCGCGCCTTCAGCGTTTTTCTGTTTGACGATCAAGGCCGTCTGCTCCTCCAACAGCGTGCCCGTAGCAAAATCACGTTCCCGAGCGTGTGGACCAACACGTGCTGCAGTCATCCGCTGCACGGCCAGACCCCAGATGAAGTGGACCAGCTGAGCCAAGTTGCGGATGGTACGGTTCCGGGCGCGAAAGCCGCCGCCATCCGCAAACTGGAACACGAACTGGGTATCCCGGCGCATCAACTGCCGGCGAGTGCGTTTCGCTTTCTGACCCGTCTGCATTACTGCGCCGCCGATGTTCAACCAGCGGCCACGCAAAGCGCCCTCTGGGGTGAACACGAGATGGACTACATTCTGTTCATCCGCGCCAACGTTACGCTGGCCCCAAACCCAGATGAGGTGGATGAAGTGCGCTACGTGACCCAAGAAGAACTGCGCCAGATGATGCAGCCGGATAACGGTCTGCAGTGGAGCCCGTGGTTTCGCATCATCGCCGCCCGCTTTCTGGAACGTTGGTGGGCGGATCTGGATGCGGCGCTGAACACCGATAAACACGAGGATTGGGGCACGGTGCACCACATCAACGAGGCCTAA

**Gene sequence of *orfA* optimized**

ATGGCGGACCGCCTGTCGGCGCGCGCGGCGATAGCCGTTGTTACGGACGATTTTACCGAATTACCGTATCCGGAGAGGACTAGCGCCCCGGATGGCCCGCTGGGTTGGCCGGGCTATGACGCCGCAAGAGCGCGTGCGGCAGAAAGGACCGGCGAACGCGAAAGCGTGATTTGCGGCACGGCGGTGGTGGAAGGCACCCGCGCGGTGGTGGTGGCGTTTGAATTTGGCTTTCTGGGCGGCAGCCTGGGCGAACGCACTGGCGACCGCCTGGAAGCGGCTTATGTGCATGCGCGCGCGCACCGCTTACCGGTGGTTCCGTTGGTAGCGACCGGCGGTAGCCGCATGCAAGAAGGTATGCTGGCTCTGACACAGCTGCAAAGGGTCGCTCGCGAAAGCGCGCTGACCCGTCAAGCGGGCCTGCCCCAAATCGCTGTGGTTAGAGACCCGACAACCGGCGGAGGCTGGGCGACCCTGGGGGCGGGCGCAGATGTCGTGTTAGCGCTTCCGGATGCGCAAGTGGGGTTCGCCGGAAGCCGTGTAAGACCGGCGGATGCAGACCCGGCGGCGTACACCGCGAGAGCACAAGTGGCGGCGGGCGCGGCGGATGCGCTGGTGCGCCCGGAAGACCTGCGCGAAACCCTGGGAAGATGGCTCCGATTGTTGACGAGACCGGCGACCGCTCCCGCGCCCCCTCCGCATGCTCTTGGGGCTGCCGGGCCACCGGCGGCGGGCTGGGACGCAGTGCGCCGCGCACGAGCGCCGGAGCGCCCGCGTGCCGCCGCGTACTTAGATGCGTATTTTACCGACCGCGTGACCCTGAGCGGCGACCGCTGCGGCGGCACCGACCCGGATGGCATGCTGTGCGGCTTTGGCGAACATGAAGGGCGGACGGTAGCATACGCAGCTCAGACGGGCACCGCTACTCGCCCGGCTGGGTATCGCACTGCGGCGAGGCTTGTGCGCCTGGCGGACCGCCTGGGAATCCCGGTGCTGACCCTGGTGGATACGCCGGGCGCGGCTAATGATGCGGAGGCGGAACGCCAAGGCGTGGGTGCAGCGATTGCGGACCTGTTTGGCGCGGTGGCTGCGGCCCGCACCCCGGTAACGACCTTAGTGGTGGGAGAAGGGGGCAGTGGCGGGGCTCTTGCTCTTGCGGCGCCGGGCAACACCTGGGTGACCCCGGATAGCTATTTTAGCGTGATTGCGCCGGAACTGGCGGCGGCAATTCTGAAGAGGCCCCCGGATGCTGTGCGCACAACGGCGGACCAACTGAGGGTGCGCCCACAAGACCTGGTCGCGCTGGGTGTCGTGCGTGGTGTGGTTGGCCCGGCGGGAGCGGGCGCGCCGCCTGGCTAA

**Gene sequence of *orfB* optimized**

ATGACAGCGGCTACCCCGGATGCCCCGCTGTGGCAGCCTGGCCCGGACCGCATTGAAGCGGCGGCGGTGACCCGGTTTCAGAATTGGGCGGCTACCCGCTATGGCGCCCCGGCGGACGGCGGCTATGCGGCGCTGCATCGCTGGAGCGTGGATGAACTGGATACCTTTTGGCGTGCGGTGGCTGAATGGTTTGATGTGCGCTTTAGCACCCCGTATGAAAGCGTGCTGGGCGACCGCGCGATGCCGGGCGCGACCTGGTTTCCCGGCGCGACCTTGAACTACGCGGAACATGCGCTGCGCACGGCGGAAGACCCGGCGCGCGCGGATACCCCGGCGCTGCTGTATGTGGATGAAACCCATACCCAACTGCCGGTGTCCTGGGCAGAATTGCGCCGTCAAGTGGGCGCACTGGCAGCGGAACTTAGAGCGCTGGGCGTGACGCCGGGCGACCGCGTGAGCGGCTATCTGCCGAACATTCCGCAAGCGGTGGTGGCGTTTCTGGCGACGGCGGCGGTGGGCGGCATTTGGACGAGCTGCGCGCCGGATTTCGGCGCGCGCTCAGTGCTGGACCGCTTTCAGCAGATTGAACCGGTGGTGCTGTTTACGGTGGATGGCTATCGCTATGGCGGCAAAGAACATGACCGCACCGAAACGGTGGCGGAACTACGTCGCGAATTACCCACCCTGAGGGCGGTGGTGCATATACCGTTACTGGGAACGGATGCCCCGGAAGGCGCGCTGGCGTGGGCGGCGCTGACGAGCGCGGATACCGAACCGGTGTTTGAACAAGTGCCGTTTGAACATCCGCTGTGGGTGCTGTATAGCAGCGGCACCACCGGCCTGCCGAAAGCGATTGTGCAGAGTCAAGGCGGCATTCTGCTGGAACATTTTAAACAGATTGGCCTGCATTGCGATTTAGGCCCGGAAGACCGCTTTTTTTGGTATACGAGCACCGGCTGGATGATGTGGAACTTTCTGGTGAGCGGCCTGCTGACCGGCACGACGGTGGTGCTGTATGATGGCAGTCCGGGCTATCCGGATGTGAGCGCGCAGTGGCGCGTGGCGGAACAGACCGGTGCTACCCTGTACGGAACGAGCGCGGCGTATGTGATGGCGTGCCGCAAAGCGGGCATTCATCCGGGCCGCGATTTTGACCTGAGCCGCGTGCAGTGCGTGGCGACCACCGGCAGCCCGCTGCCGCCGGATGGCTTTCGCTGGCTGCATGATGAAGTGGCGGAAGACCTGTGGATTGCGAGCGTGAGCGGCGGCACCGATGTGTGCAGCTGCTTTGCGGGCGCGGTGCCGACCCTGCCGGTGCATATTGGCGAACTGCAAGCGCCCTGCCTTGGCACCGACTTACAGAGCTGGGACCCGGCGGGCCGCCCGCTGATTGGCGAAGTGGGCGAACTGGTGGTGACCAACCCGCTGCCGAGCATGCCGATTCGCTTTTGGAACGACCCGGATGGCAGCCGCTATCATGATAGCTATTTTGATGTGTATCCGGGCGTGTGGCGTCATGGCGATTGGATTACCCTGACCGACCGGGGCAGCGTGATTATTCATGGCCGCAGCGATAGCACCCTGAACCGCCAAGGCGTGCGCATGGGCAGCGCGGATATTTATGAAGCGGTGGAACGCCTGCCGGAAATTCGCGAAAGCCTGGTGATTGGCCTGGAAGAACCGGATGGCGGGTATTGGATGCCGCTGTTTGTGCATCTGGCGGATGGAGCCACCCTGGATGATGACCTGCGCGATGCGATTAAACGCACCATTCGCGAAAACCTGAGCCCGCGCCATGTGCCGGATGAAGTGATTGAAGTTCCGGCGATTCCGCATACCCTGACCGGCAAACGCATTGAAGTACCCGTGAAGCGCCTGCTGCAAGGCACCGAACTGGCGAAAGCGGTGAATCCGGGCAGCGTGGATAACCTGGACCTGCTGCATTTTTATGCGGAACTGGCGGCGAGCCGCCGCCGCTAA

**Gene sequence of *orfC* optimized**

ATGGCGAGCGATAGCGGCATTGGCATTCATGACCTGAGCTTTGCGACCACCGAATTTGTGCTGCCGCATACCGCGCTGGCGGAATATAACGGCACCGAAATTGGCAAATATCATATTGGCATTGGACAAGAAAGCATGAGCGTTCCGGCGGCGGATGAAGATATTGTGACCCTGGGAGCGGCTGCGGCGGCGCCGATTATTGAACGTCATGGCAGCGAACGCATTCGCACGGTGGTGTTTGCGACCGAAAGCAGCATTGACCAAGCGAAAAGCGGCGGCGTGTATGTGCATAGTCTGCTGGGCCTGCCGAGCGCGGCGCGCGTGGTGGAACTGAAACAAGCGTGCTACGGAGCGACGGCGGGTCTTCAGTTCGCACTGGGCCTGGTGGCGCGCGACCCGAGTCAGCAAGTGCTGGTGATTGCGAGCGATGTGAGCAAATATGAATTAGATAGCCCTGGAGAAGCCACCCAAGGCGCGGCGGCGGTGGCGATGCTGGTGGGCGCGGACCCGCAGCTGATTCGCTTTGATGGTCCGAGCGGCCTGTTTACGGCGGATGTGATGGATTTTTGGCGCCCGAACTATCGCGATGCGGCGCTGGTGGATGGTCAAGAAAGCATTAGCGCGTATCTGCAAGCGGTGGAAGGCTGCTGGAAAGATTATGCGGAACAAGGCGGCCATAGCCTGGATGAATTTGCGGCGTTTTGCTATCATCAGCCGTTTACCAAAATGGCGTATAAAGCGCATCGCCATCTGCTGGAATATTGCGGCCATGATAGCGATAAAGATGCGGTGGAACAAGCGCTGGGTCAGACCACCGCGTATAACCGCGTGATTGGCAACAGCTATACCGCGAGCGTGTATCTGGGCTTAGCGGCGCTGCTGGACCAAGCGGATGACCTGACCGGCCGCAGCATTGGCTTTCTGAGCTATGGCAGCGGCAGCGTGGCGGAATTTTTTGCGGGCCAAGTGGTGCCGGGGTATAGGGACCGCCTGCGCACGGCGGAACATCGCAAAGCGGTGGAACGCCGCACCGTGCTGGATTATGCGGGCTATCGCGAACTGCATGAACGCAAATTTCCGACCGATGGCGGCGATTATCCGACCCCGGTGCAGAGCGGCGGTCAGTTTCGCCTGGCGGCGCTGCGTGGCCATAAACGCATTTATGAACGCCGCTAA

**Gene sequence of *orfD* optimized**

ATGACCGAAACCCATGCTATCGCGGGCGTGCCGATGCGCTGGGTTGGCCCGATTCGTATCAGCGGCAACGTGGCCACCACAGAAACCCACGTACCCCTTGCGACATACGAAACCCCGCTGTGGCCGAGCGTCGGTCGCGGCGCGAAAGTGAGCCGCCTGGTGCAAGAAGGCATTGTAGCGACATTGGTGGATGAACGCATGACCCGCAGCGTGCTGGTGGAAGCGACCGATGCGCAGACCGCGTATGTGGCGGCGCGCGCGATTGATGCGCGCATTGATGAACTGCGCGAAGTGGTGCGCGGTTGCAGCCGCTTTGCGCAGCTGATTAACATTCGCCATGAAATTAACGCGAACCTGCTGTTTATTCGCTTTGAATTTACCACCGGCGATGCGAGCGGCCATAACATGGCGACGCTTGCGAGCGATGCGCTGCTGAAACATCTGCTGGAAACCATTCCGGGCATTAGCTATGGCAGCATCAGCGGGAACTACTGTACCGATAAAAAAGCGACCGCGATTAACGGCATTCTGGGTCGCGGCAAAAACGTGGTGACCGAACTGCTGATTCCGCGCGATATTGTGACCGATGTGCTGCATACGACGGCGGCGAAAGTGGTGCAGCTGAACATTCGCAAAAACATGCTGGGCACCCTGCTGGCGGGCGGCATTCGCAGCGCGAACGCGCATTATGCGAACATGCTGCTGGGCTTTTATCTGGCGACCGGCCAAGATGCGGCGAACATTGTGGAAGGCAGCCAAGGCGTGACGATGGCGGAAGACCGCGATGGCGACCTGTATTTTGCGTGCACCCTGCCGAACCTGATTGTGGGCACGGTGGGCAACGGCAAAGGCCTGGAGTTTGTAGAAACGAACTTAGCCCGCTTAGGGTGCCGCGCGGATAGAGAACCGGGCGAAAACGCTCGCCGCCTGGCAGTGATTGGCGCGGCGACCGTGCTGTGCGGCGAACTGAGCCTGCTGGCGGCGCAGACCAATCCGGGCGAACTGATGCGCGCGCATGTGCAGCTGGAACGCGGCAACAAAACCGAAAAAGTGGGCGTGTAA

**Gene sequence of *hmgr* optimized**

ATGCAGAAACGTCAGCGCGAACTGAGCGCGCTGACCCTGCCGACGAGCGCGGAAGGCGTGAGCGAAAGCCATCGCGCGCGCAGCGTGGGCATTGGCCGCGCGCATGCGAAAGCGATTCTGCTGGGGGAACATGCTGTAGTGTACGGCGCCCCGGCGTTAGCCCTGCCCATCCCACAGCTGACCGTGACCGCGAGCGTGGGCTGGAGCAGCGAAGCGAGCGATAGCGCGGGCGGCCTGAGCTATACCATGACCGGCACCCCGAGCCGCGCGCTGGTGACCCAAGCGAGCGATGGCCTGCATCGCCTGACGGCGGAATTTATGGCGCGCATGGGCGTGACCAACGCGCCGCATCTGGATGTTATTCTGGATGGGGCGATACCGCATGGCCGAGGGCTAGGCAGCAGCGCGGCGGGAAGTCGTGCGATTGCGCTGGCTCTGGCCGACCTGTTTGGCCATGAACTGGCGGAACATACCGCGTATGAACTGGTGCAGACGGCGGAAAACATGGCACATGGCCGCGCGAGCGGCGTGGATGCGATGACGGTGGGCGCGAGCCGCCCGCTGCTGTTTCAGCAAGGCCGCACCGAACGCCTGGCGATTGGCTGCGATAGCCTGTTTATTGTGGCGGATAGCGGCGTGCCGGGCAGCACCAAAGAAGCGGTGGAAATGCTGCGCGAAGGCTTTACCCGCAGCGCGGGCACCCAAGAACGCTTTGTCGGAAGAGCCACCGAACTGACCGAAGCAGCGCGTCAAGCGCTGGCGGATGGCCGCCCGGAAGAACTGGGCAGTCAGCTGACCTATTATCATGAACTGCTGCACGAAGCGCGCCTGAGCACCGATGGCATTGATGCGCTGGTGGAAGCGGCGCTGAAAGCGGGCAGCCTGGGCGCGAAAATTACCGGCGGCGGTCTGGGCGGCTGCATGATTGCGCAAGCGCGCCCGGAACAAGCGCGCGAAGTGACCCGTCAGCTGCATGAAGCGGGCGCGGTGCAGACCTGGGTGGTGCCGCTGAAAGGCCTGGATAACCATGCGCAGTAA

**Gene sequence of *orfE* optimized**

ATGAGCGAACAGCAAATTGCTGTCCTGCGCCCGCGCGAACAGCCGGGGGCGAACGGAGTGACGGCGGTGGCGCAGCCGAACATTGCGCTGATTAAATATTGGGGCAAACGCGATGAACATCTGTTTCTGCCTTGGACGAGCAGCCTGAGCATGACCCTGGATATTTTTCCGACCACCACCCGCGTGCATTTAGATGCGGAAGCGACCGATGATGAAGTGACCTTTAACGGCGCTCCGGCGGCGGGCGAGGAGCGCCGCCGCATTACCGGCTTTCTGGACCTGGTGCGTCAGCGCGCGGGCCTGACACATCGTGCGGTTGTTGATACAAGAAATACCGTGCCAACCGGCGCGGGCCTGGCGTCGAGCGCGGGTGGGTTTGCGGCGCTGGCGGTGGCGGCGGCGACCGCGTATGGATTAGACCTGGATGACACCGGCTTAAGCCGCCTGGCGCGCAGAGGCAGCGGCAGCGCGAGCCGCAGCATTTTCGGTGGGTTTGCAGTGTGGAATGCGGGAACCCCGACAGCGCCACCGGCGGAAGCGGACCTGAGCAGCTATGCGGAACCGGTGCCGGTGGGCGACCTGGACCCGGCGCTGGTGATTGCGGTGGTGAACGCTGGCCCGAAAGATGTGAGCAGCCGCGCGGCGATGCGCCGCACGGTGGAAACGAGCCCGCTGTTTGAACCTTGGGCGGCGAGCAGCCGCGATGACCTGACCGAAATGCGCCAAGCGCTGCTGCGCGCGGACCTGGATGCGGTGGGCGAAATTGCGGAACGCAACAGCCTGGGCATGCATGCGACCATGCTAAGCGCGCGCCCTGCGGTGCGCTATCTGAGCCCGGCGAGCCTGACCGTGCTGGATAGCGTGCTGCGCCTGCGTCGCGATGGCATTAGCGCGTATGCGACGATGGATGCTGGCCCGAACGTGAAGGTGCTGTGTCGCCGCGCGGATGCGGACTTAGTGGCGGAAGCGGTGCGCGCGGCAGCGGCGGGCGGCACAGTGCATATTGCGGGCACCGGCCAAGGCGCGCGCCTGATTGATGAAGATGGCCGCTAA

**Gene sequence of *ispD***

ATGGCAACCACTCATTTGGATGTTTGCGCCGTGGTTCCGGCGGCCGGATTTGGCCGTCGAATGCAAACGGAATGTCCTAAGCAATATCTCTCAATCGGTAATCAAACCATTCTTGAACACTCGGTGCATGCGCTGCTGGCGCATCCCCGGGTGAAACGTGTCGTCATTGCCATAAGTCCTGGCGATAGCCGTTTTGCACAACTTCCTCTGGCGAATCATCCGCAAATCACCGTTGTAGATGGCGGTGATGAGCGTGCCGATTCCGTGCTGGCAGGTCTGAAAGCCGCTGGCGACGCGCAGTGGGTATTGGTGCATGACGCCGCTCGTCCTTGTTTGCATCAGGATGACCTCGCGCGATTGTTGGCGTTGAGCGAAACCAGCCGCACGGGGGGGATCCTCGCCGCACCAGTGCGCGATACTATGAAACGTGCCGAACCGGGCAAAAATGCCATTGCTCATACCGTTGATCGCAACGGCTTATGGCACGCGCTGACGCCGCAATTTTTCCCTCGTGAGCTGTTACATGACTGTCTGACGCGCGCTCTAAATGAAGGCGCGACTATTACCGACGAAGCCTCGGCGCTGGAATATTGCGGATTCCATCCTCAGTTGGTCGAAGGCCGTGCGGATAACATTAAAGTCACGCGCCCGGAAGATTTGGCACTGGCCGAGTTTTACCTCACCCGAACCATCCATCAGGAGAATACATAA

**Gene sequence of *ispF***

ATGCGAATTGGACACGGTTTTGACGTACATGCCTTTGGCGGTGAAGGCCCAATTATCATTGGTGGCGTACGCATTCCTTACGAAAAAGGATTGCTGGCGCATTCTGATGGCGACGTGGCGCTCCATGCGTTGACCGATGCATTGCTTGGCGCGGCGGCGCTGGGGGATATCGGCAAGCTGTTCCCGGATACCGATCCGGCATTTAAAGGTGCCGATAGCCGCGAGCTGCTACGCGAAGCCTGGCGTCGTATTCAGGCGAAGGGTTATACCCTTGGCAACGTCGATGTCACTATCATCGCTCAGGCACCGAAGATGTTGCCGCACATTCCACAAATGCGCGTGTTTATTGCCGAAGATCTCGGCTGCCATATGGATGATGTTAACGTGAAAGCCACTACTACGGAAAAACTGGGATTTACCGGACGTGGGGAAGGGATTGCCTGTGAAGCGGTGGCGCTACTCATTAAGGCAACAAAATGA

**Efficient synthesis of cembratriene-ol via systematic optimization in this study**


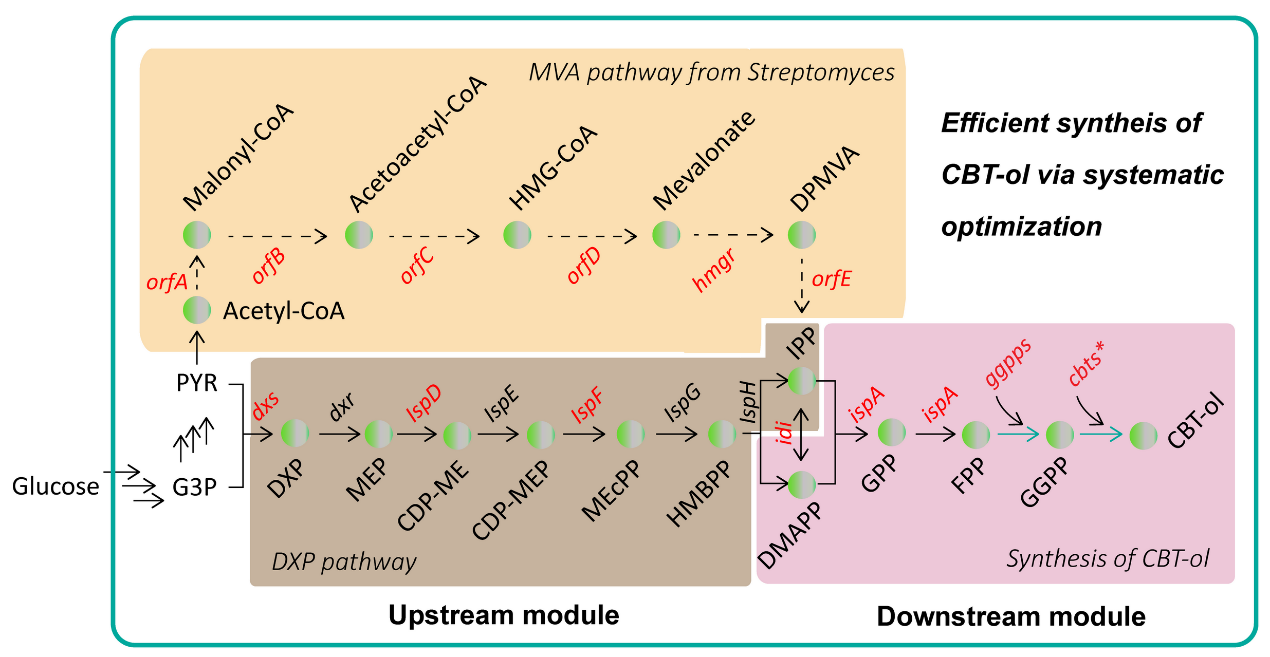


**Fig. S1** Efficient synthesis of cembratriene-ol via systematic optimization in this study. *ggpps*, geranylgeranyl pyrophosphate synthase gene; *cbts**, lacked a plastid transit peptide (52 AA, 156 bp) compared with cembratriene-ol synthase gene *cbts*. Genes *orfA*, *orfB*, *orfC*, *orfD*, *hmgr*, and *orfE* from MVA pathway of *Streptomyces* encoding acetyl-CoA carboxylase, acetoacetyl-CoA synthase, hydroxymethylglutaryl-CoA synthase, hydroxymethylglutaryl-CoA reductase, mevalonate kinase, and mevalonate diphosphate decarboxylase, respectively. HMG-CoA, 3-hydroxy-3-methyl glutaryl coenzyme A; DPMVA, diphosphomevalonate.

**Gas chromatographic (GC) profile and the associated mass peaks of cembratriene-ol**


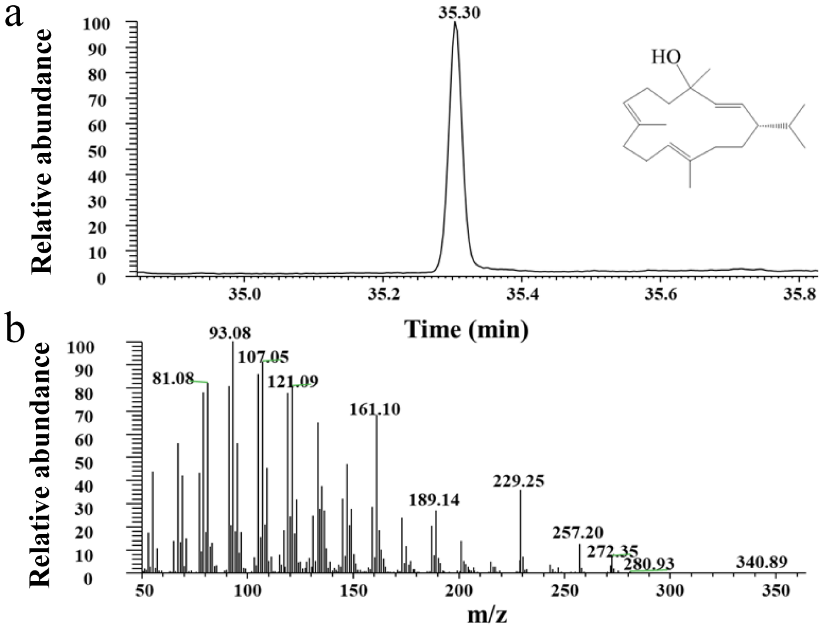


**Fig. S2** GC profile and the associated mass peaks of cembratriene-ol. a, GC profile. b, The associated mass peaks. The associated mass peaks of cembratriene-ol was *m*/*z* 229.25.

**Effects of expression of cembratriene-ol synthase gene *cbts* on growth of recombinant *E. coli***


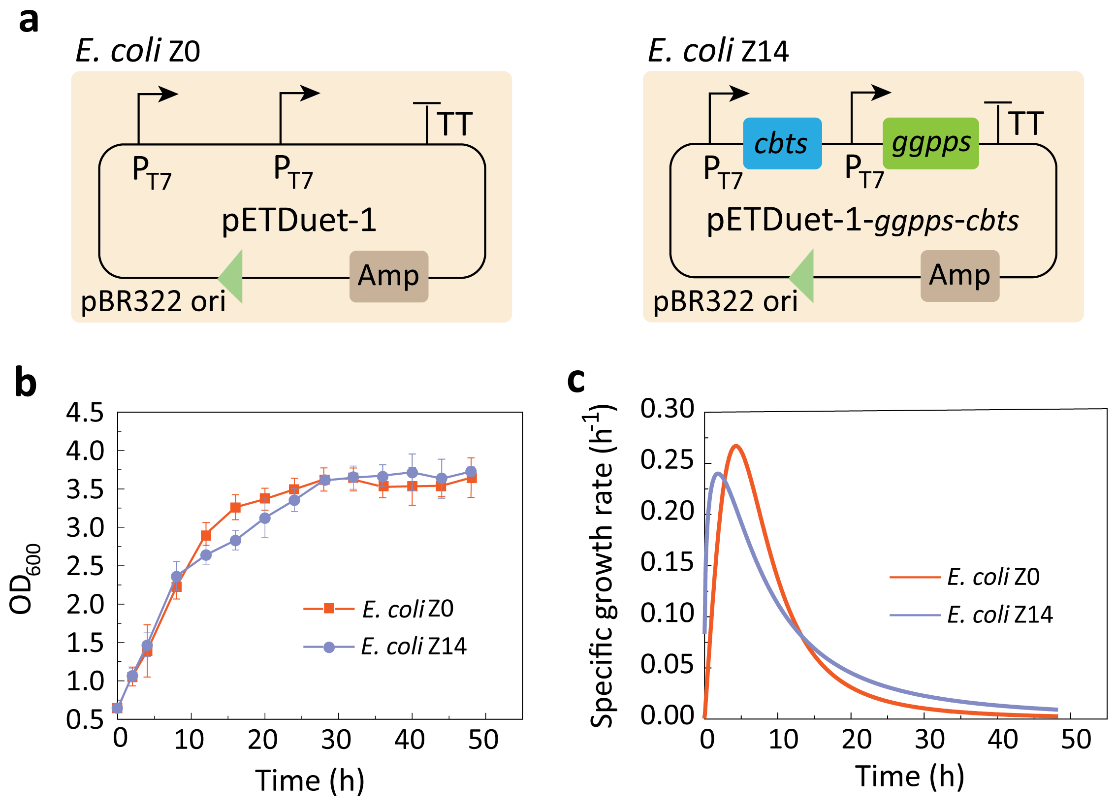


**Fig. S3** Effects of expression of cembratriene-ol synthase gene *cbts* on growth of recombinant *E. coli*. a, Schematic diagram of the construction of recombinant strains. b, Growth curve. c, Specific growth rate.

**The residual glucose concentrations of different strains**


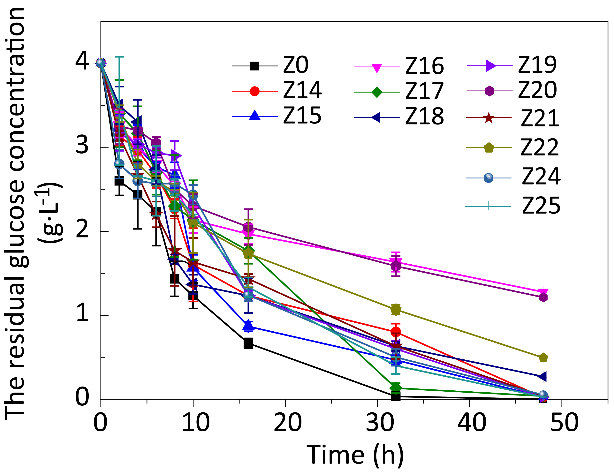


**Fig. S4** The residual glucose concentrations of different strains

**Effects of expression of the cembratriene-ol synthase truncated variant gene *cbts** on growth of recombinant *E. coli***


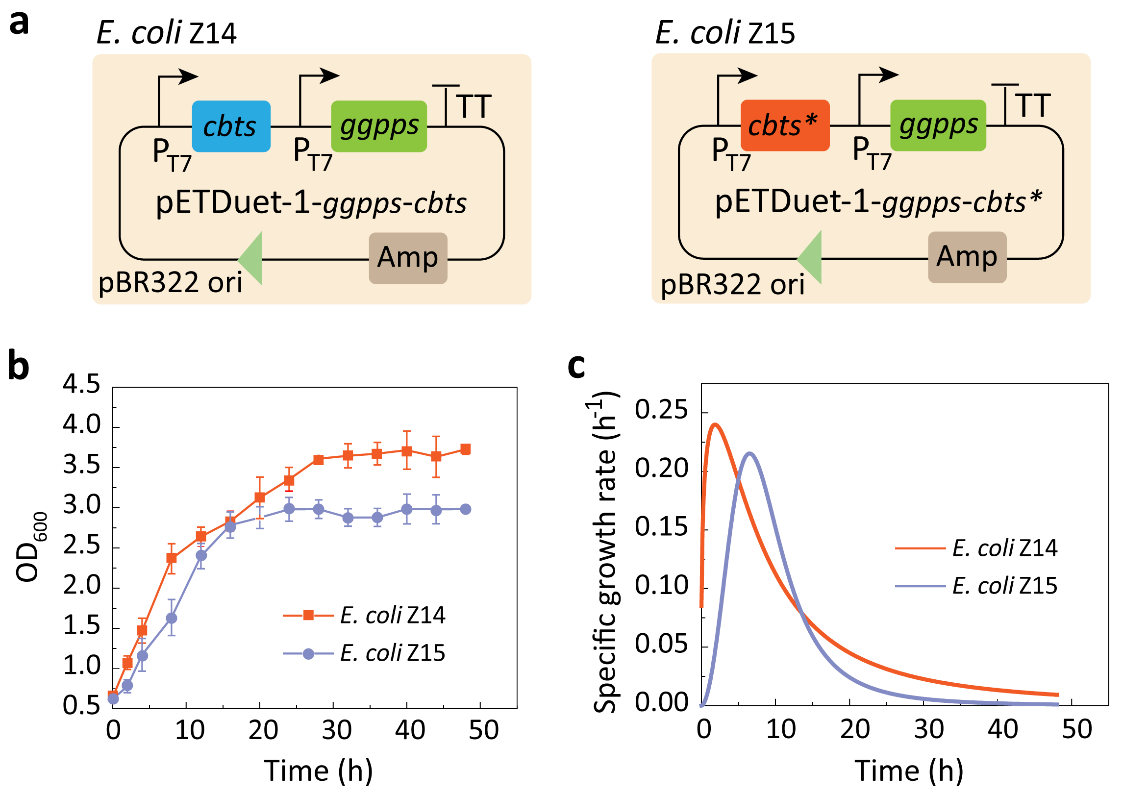


**Fig. S5** Effects of expression of the cembratriene-ol synthase truncated variant gene *cbts** on growth of recombinant *E. coli*. a, Schematic diagram of the construction of recombinant strains. b, Growth curve. c, Specific growth rate.

**SDS-PAGE of CBTS and CBTS* expressed in *E*. *coli***

Enzyme CBTS* solubility was reduced compared with CBTS with the plastid transit peptide when expressed with plastid transit peptide sequence (Fig. S6).


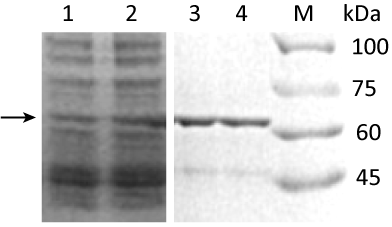


**Fig. S6** SDS-PAGE of CBTS and CBTS*. 1, The supernatant of *E*. *coli* Z14 lysed. 2, The supernatant of *E*. *coli* Z15 lysed. 3, The precipitation of *E*. *coli* Z14 lysed. 4, The precipitation of *E*. *coli* Z15 lysed. M, Standard molecular weight proteins. Arrow, The target enzymes CBTS or CBTS*. *E*. *coli* Z14, expression of CBTS. *E*. *coli* Z15, expression of CBTS*. OD_600_ of *E*. *coli* Z14 and *E*. *coli* Z15 lysed was 1.7.

**Effects of overexpression of *dxs* and *idi* on growth of recombinant *E. coli***


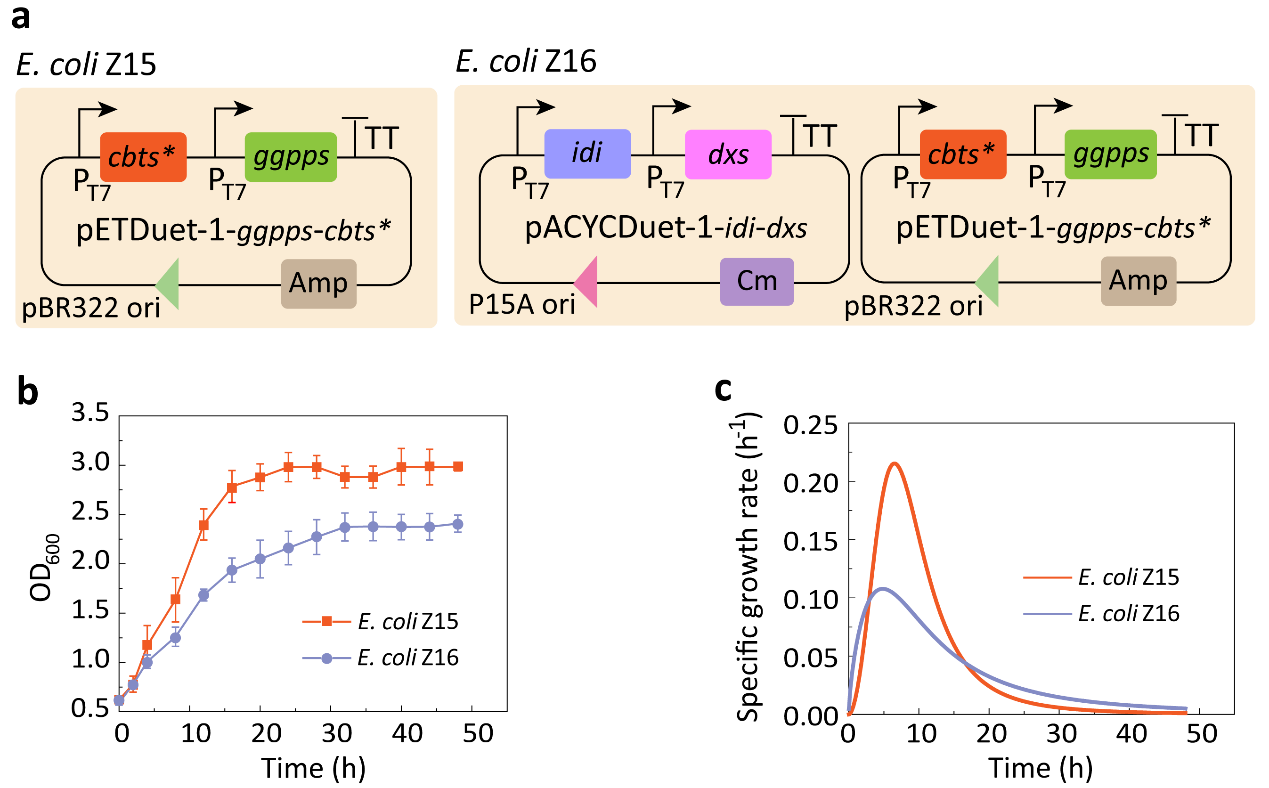


**Fig. S7** Effects of overexpression of *dxs* and *idi* on growth of recombinant *E. coli*. a, Schematic diagram of the construction of recombinant strains. b, Growth curve. c, Specific growth rate.

**Effects of overexpressing key genes on growth of recombinant *E. coli***


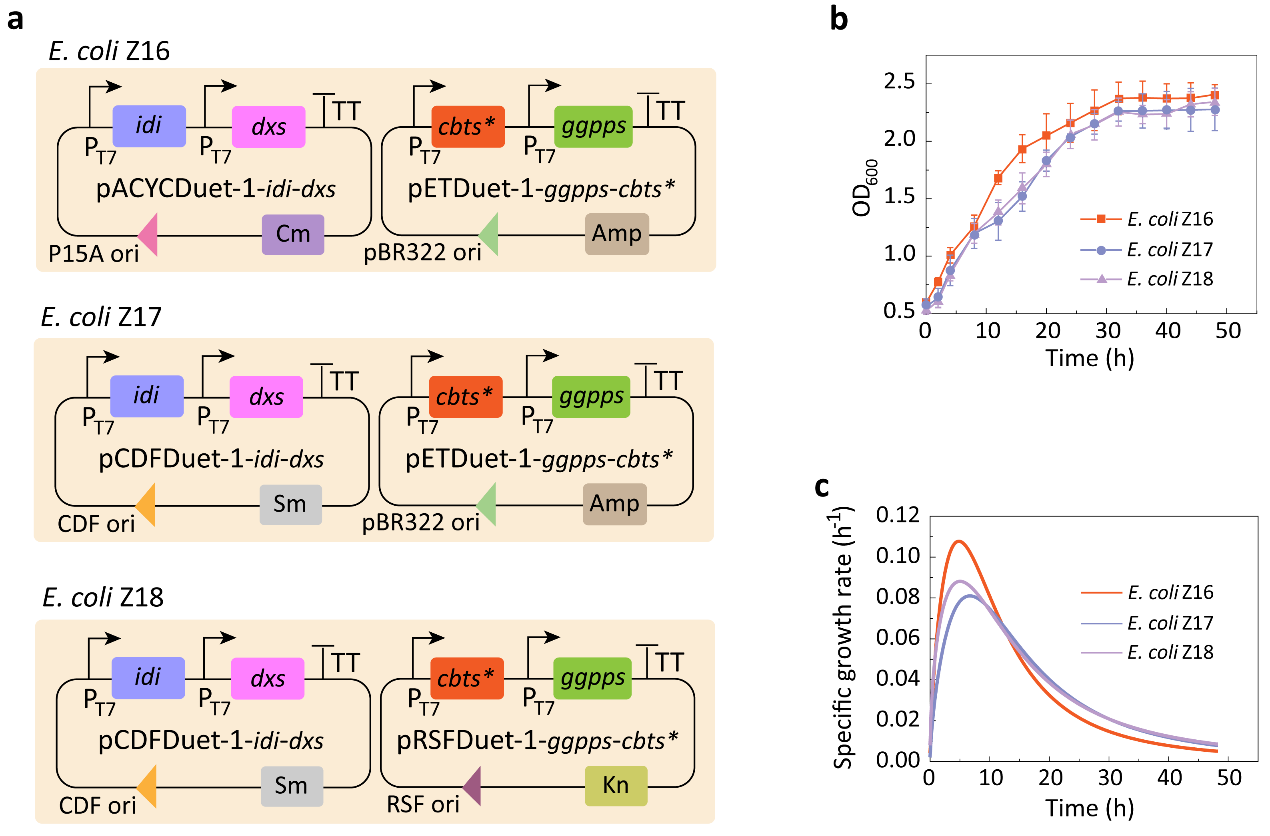


**Fig. S8** Effects of overexpressing key genes on growth of recombinant *E. coli*. a, Schematic diagram of the construction of recombinant strains. b, Growth curve. c, Specific growth rate.

**Effects of systematic regulating the expression level of key genes** **on growth of recombinant *E. coli***


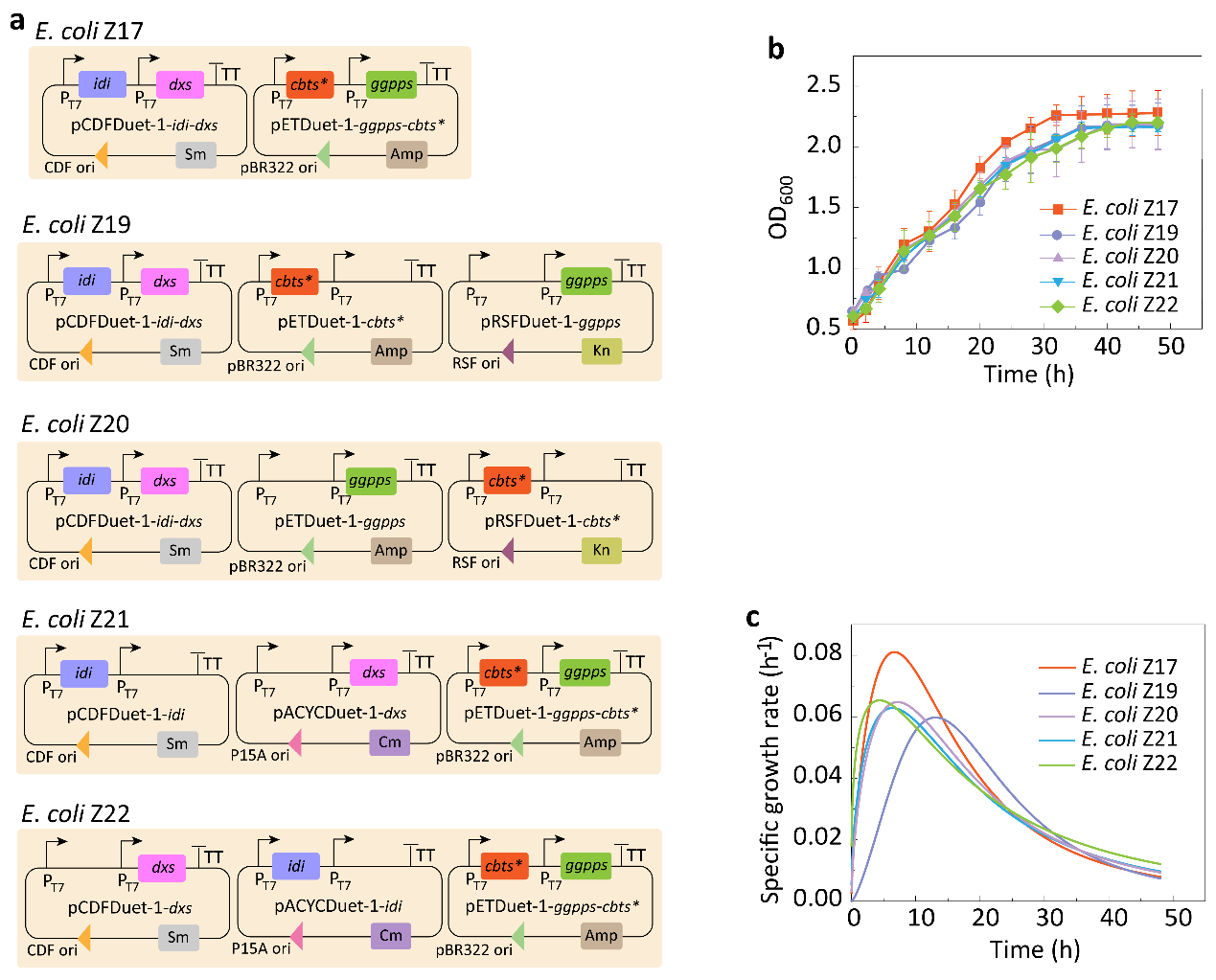


**Fig. S9** Effects of systematic regulating the expression level of key genes on growth of recombinant *E. coli*. a, Schematic diagram of the construction of recombinant strains. b, Growth curve. c, Specific growth rate.

**Determination of plasmid copy numbers by Quantitative Real-time PCR (qPCR)**

The qPCR and total DNA extracts were used to determine plasmid copy numbers. E. coli cells were grown in initial fermentation medium. The fermentation conditions for producing **c**embratriene-ol in 250 mL flasks (75 mL fermentation media) were a 1% (v/v) inoculum, 30 °C, 200 rpm, and 150 μM IPTG (final concentration) and lowering the fermentation temperature to 22 °C until OD_600_ = 0.6. After 48 h, cells were collected at 12,000 ×*g* at 4 °C for 1 min, and the total DNA was extracted. The resistance genes on the plasmids were selected as the target genes: pRSFDuet-1, kanamycin resistance gene; pETDuet-1, ampicillin resistance gene; pCDFDuet-1, streptomycin/spectinomycin resistance gene; pACYCDuet-1, chloramphenicol resistance gene. The orotidine-5'-phosphate decarboxylase gene *pyrF* on the chromosome of *E*. *coli* was selected as the reference gene. Copy numbers were quantified by qPCR on the Applied Biosystems StemOnePlus^TM^ (Thermo Scientific). The primers for qPCR were *pyrF*-FW/ *pyrF*-RS for *pyrF* (Table S3). In this study, the actual copy numbers of empty plasmids pACYCDuet-1, pCDFDuet-1, pETDuet-1, and pRSFDuet-1 are 3.38, 4.89, 9.12, and 26.3 at the cultivation conditions, respectively (Fig. S10).


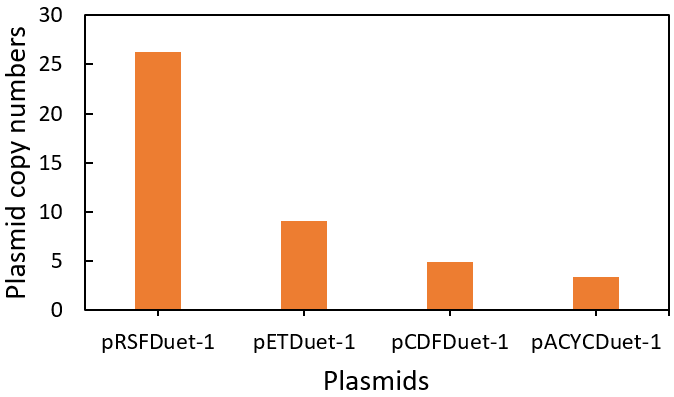


**Fig. S10** The copy numbers of different plasmids at the cultivation conditions in this study

**Effects of overexpression of gene *ispA* on growth of recombinant *E. coli***


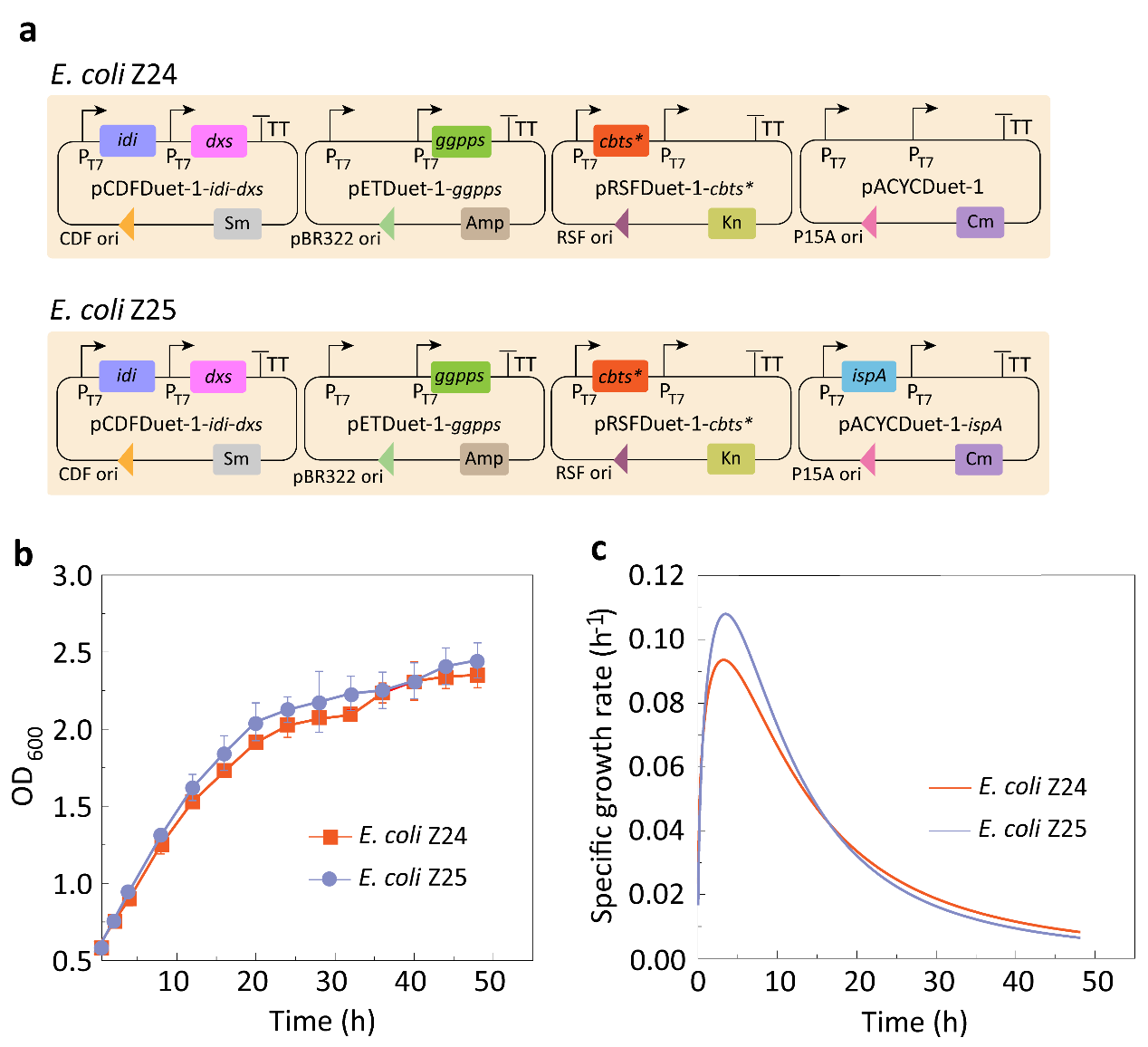


**Fig. S11** Effects of overexpression of gene *ispA* on growth of recombinant *E. coli*. a, Schematic diagram of the construction of recombinant strains. b, Growth curve. c, Specific growth rate.

**Effects of fermentation optimization on growth of recombinant *E. coli***


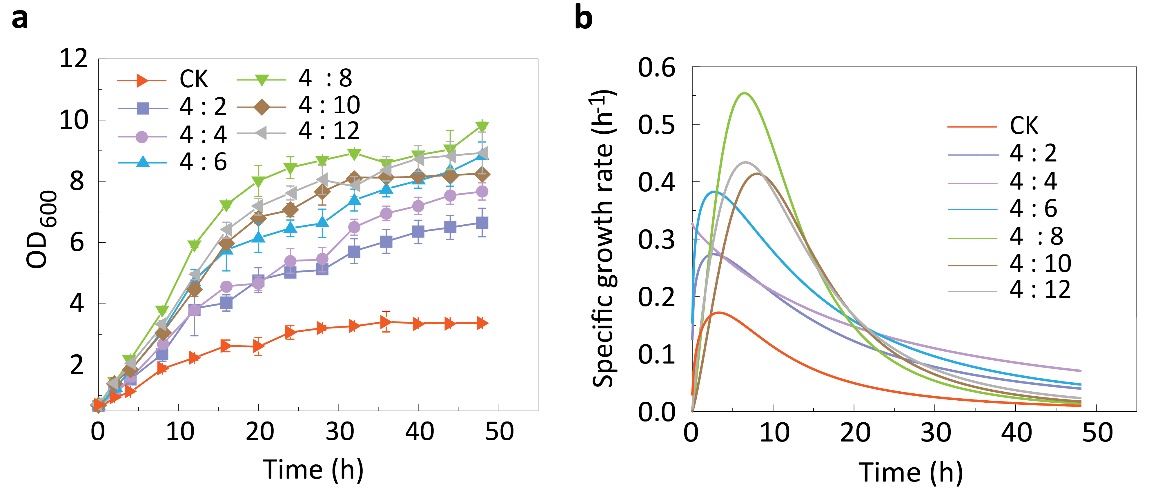


**Fig. S12** Effects of fermentation optimization on growth of recombinant *E. coli*. a, Growth curve. b, Specific growth rate.

**Effects of introduction of the heterologous MVA pathway on the glucose utilization, conversion yield, and growth of recombinant *E. coli***

**
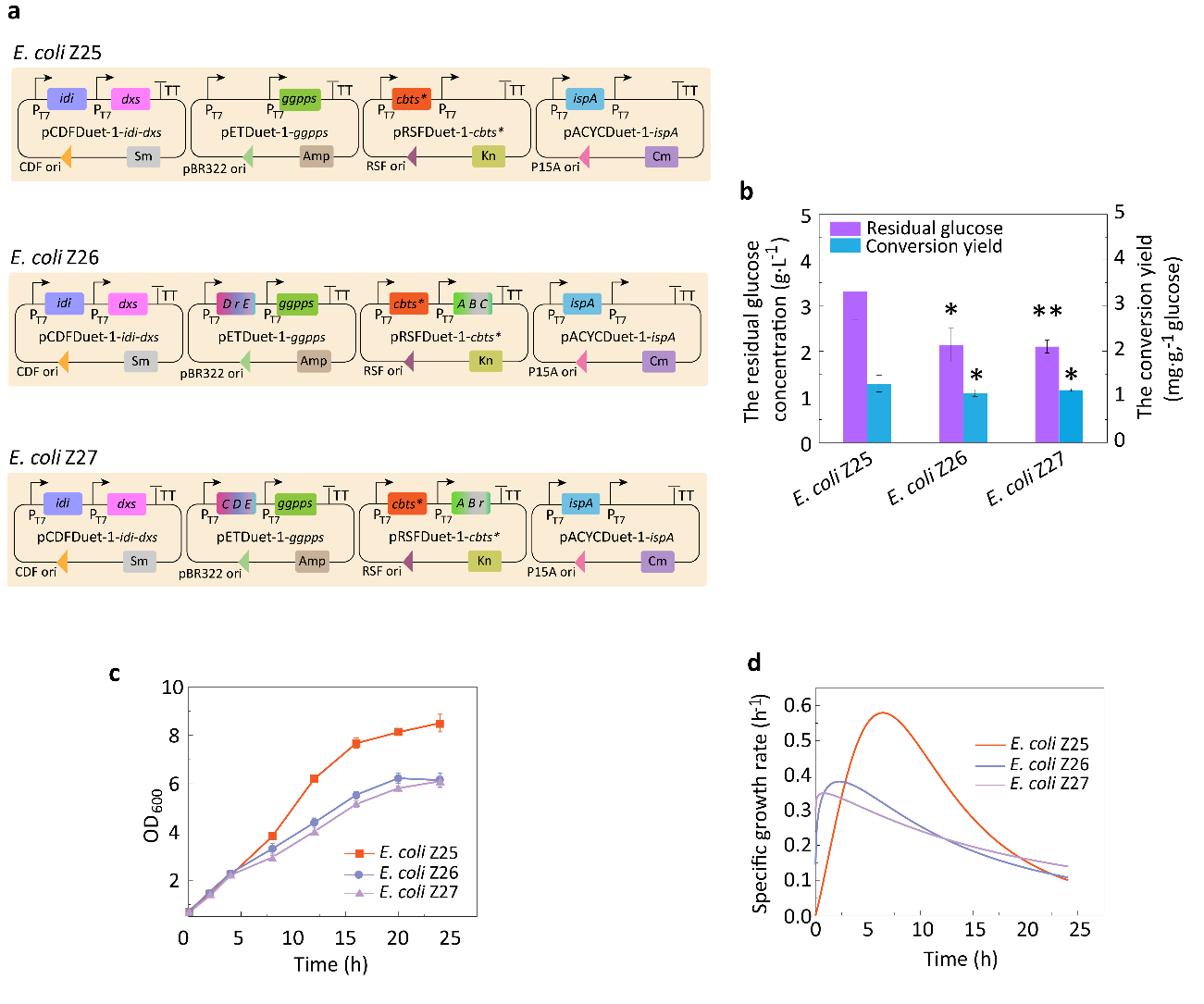
**

**Fig. S13** Effects of introduction of the heterologous MVA pathway on the glucose utilization, conversion yield, and growth of recombinant *E. coli*. a, Schematic diagram of the construction of recombinant strains. *ABC*, *orfA*, *orfB*, and *orfC*; *DrE*, *orfD*, *hmgr*, and *orfE*; *ABr*, *orfA*, *orfB*, and *hmgr*; *CDE*, *orfC*, *orfD*, and *orfE*. b, Residual glucose concentration and conversion yield of glucose to cembratriene-ol at 24 h. Student’s *t*-test was used to statistically analyze data, and statistical significance was set at *p* value < 0.05. Asterisks indicate significant differences compared to *E*. *coli* Z25 (**, *p* < 0.05; *, *p* > 0.05). *p* < 0.05 was considered statistically significant. c, Growth curve. d, Specific growth rate.

**The concentrations of lactic acid and acetic acid in *E*. *coli* Z26 and *E*. *coli* Z27**


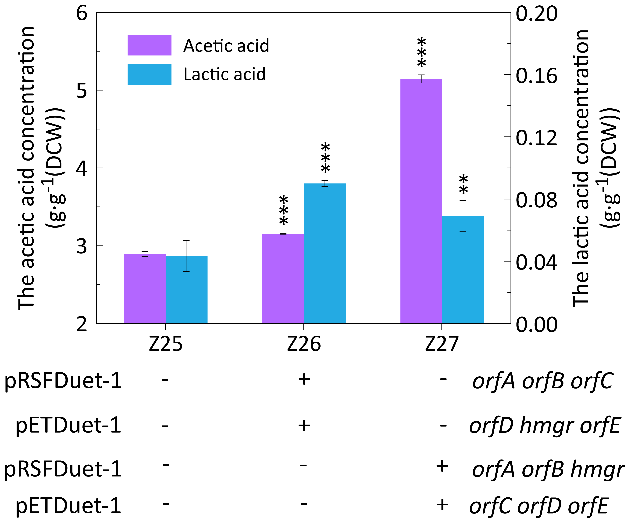


**Fig. S14** The concentrations of lactic acid and acetic acid in *E*. *coli* Z26 and *E*. *coli* Z27. Student’s *t*-test was used to statistically analyze data, and statistical significance was set at *p* value < 0.05. Asterisks indicate significant differences compared to *E*. *coli* Z25 (***, *p* < 0.01; **, *p* < 0.05). *p* < 0.05 was considered statistically significant.
